# cDCBLD2 mediates sorafenib resistance in hepatocellular carcinoma by sponging miR-345-5p binding to the TOP2A coding sequence

**DOI:** 10.7150/ijbs.86227

**Published:** 2023-08-28

**Authors:** YeLing Ruan, TianYi Chen, LongBo Zheng, JingWei Cai, Hu Zhao, YaLi Wang, LiYe Tao, JunJie Xu, Lin Ji, XiuJun Cai

**Affiliations:** 1 Key Laboratory of Laparoscopic Technology of Zhejiang Province, Department of General Surgery, Sir Run-Run Shaw Hospital, Zhejiang University School of Medicine - Hangzhou, China.; 2 Zhejiang Minimal Invasive Diagnosis and Treatment Technology Research Center of Severe Hepatobiliary Disease, Zhejiang Research and Development Engineering Laboratory of Minimally Invasive Technology and Equipment - Hangzhou, China.; 3 Zhejiang University Cancer Center - Hangzhou, China.; 4 Liangzhu Laboratory, Zhejiang University Medical Center - Hangzhou, China.; 5 Department of Gastroenterology, The Affiliated Hospital of Qingdao University - Qingdao, China

**Keywords:** HCC, sorafenib, circRNA, miRNA345-5p, TOP2A

## Abstract

Sorafenib is a first-line chemotherapy drug for treating advanced hepatocellular carcinoma (HCC). However, its therapeutic effect has been seriously affected by the emergence of sorafenib resistance in HCC patients. The underlying mechanism of sorafenib resistance is unclear. Here, we report a circular RNA, cDCBLD2, which plays an important role in sorafenib resistance in HCC. We found that cDCBLD2 was upregulated in sorafenib-resistant (SR) HCC cells, and knocking down cDCBLD2 expression could significantly increase sorafenib-related cytotoxicity. Further evidence showed that cDCBLD2 can bind to microRNA (miR)-345-5p through a competing endogenous RNA mechanism, increase type IIA topoisomerase (TOP2A) mRNA stability through a miRNA sponge mechanism, and reduce the effects of sorafenib treatment on HCC by inhibiting apoptosis. Our findings also suggest that miR-345-5p can negatively regulate TOP2A levels by binding to the coding sequence region of its mRNA. Additionally, targeting cDCBLD2 by injecting a specific small interfering RNA (siRNA) could significantly overcome sorafenib resistance in a patient-derived xenograft (PDX) mouse model of HCC. Taken together, our study provides a proof-of-concept for a potential strategy to overcome sorafenib resistance in HCC patients by targeting cDCBLD2 or TOP2A.

## Background

Liver cancer is one of the most common cancers and the third leading cause of cancer-related deaths worldwide 1. Hepatocellular carcinoma (HCC) accounts for nearly 90% of all primary liver cancer cases. About half of HCC patients receive systematic treatment during their disease course, especially in the advanced stage 2. Among HCC treatments, the most well-known targeted drug is the multi-target tyrosine kinase inhibitor sorafenib, which is an inhibitor of the Raf/MEK/ERK pathway. It was the first clinically targeted drug approved by the United States Food and Drug Administration (FDA) for treating advanced HCC. Sorafenib is the current first-line treatment option for advanced HCC 3. However, sorafenib has displayed indications of drug resistance during long-term clinical application, which can seriously limit its clinical therapeutic effect. The specific drug resistance mechanism has not been fully clarified. Developing new drugs is an expensive process, and the average drug development cycle from preclinical target screening to final marketing is at least 13.5 years (excluding the target confirmation stage) 4. Drug resistance is a key issue that limits the clinical application of targeted drugs. Addressing this problem will significantly improve the clinical application benefit ratio of relevant targeted drugs.

Many studies have investigated the mechanism of sorafenib resistance. At present, it is believed that sorafenib drug resistance can be divided into congenital drug resistance and acquired drug resistance. Cancer patients with mutations in the sites of action, such as EGFR, RAS/RAF/MEK/ERK, and VEGFR, may have innate resistance to sorafenib. This phenomenon also occurs in the newly reported resistance mechanism of Lenvatinib 5. The mechanism of acquired drug resistance is related to signaling pathways that mediate cell survival, such as PI3K/AKT, IGF/FGF, NF-κB, and TGFβ, as well as to many epigenetic factors. Tumor microenvironment (TME) factors, such as autophagy, metabolism, and immunity, are also involved, making the mechanism of sorafenib resistance even more complex 6. The regulatory roles of non-coding RNAs (ncRNAs) in tumor drug resistance mechanisms have recently attracted substantial attention. Examples of ncRNAs, long non-coding RNAs (lncRNAs) and microRNAs (miRNAs), participate in sorafenib resistance by inducing autophagy and the epithelial-mesenchymal transition in HCC cells, activating the PI3K/AKT pathway, promoting liver cancer stem cell proliferation, and regulating the TME and other biological activities 7.

Circular RNAs (circRNAs) are a type of ncRNA characterized by a ring structure. While studying the mechanism of sorafenib resistance, researchers also found that circRNAs participate in cellular events through multiple mechanisms. Among them, one in-depth study determined that circRNAs can act as a miRNA sponge by adsorbing miRNA molecules and preventing them from regulating their targets (ceRNA mechanism, competing endogenous RNA mechanism). Thomas Hansen and colleagues first proposed the functional miRNA sponge model. They identified 63 miR-7 binding sites in CDR1as, a circRNA that inhibits the actions of miR-7 8. Numerous studies have subsequently demonstrated that many circRNAs can function as miRNA sponges. CircRNAs can also bind to protein molecules, making this an additional widely recognized and accepted mode of action. Studies have found that circRNA-SORE (circRNA_104797) plays a part in the occurrence and development of sorafenib resistance by binding to Y-box binding protein 1 (YBX-1) protein 9. In the past, circRNAs were believed to be solely ncRNAs. However, recent work has found that some circRNAs can also be translated into proteins. Additionally, N6-methyladenosine (m6A) modifications have been identified in circRNA molecules, and this modification can reportedly promote protein translation from circRNAs 10.

MiRNAs are the most deeply studied type of ncRNA. They can negatively regulate gene expression by inhibiting translation or promoting mRNA degradation and have been found to be involved in sorafenib resistance of HCC 11-13. As an important molecule in post-transcriptional regulation, miRNAs can affect the development of sorafenib resistance through various mechanisms. Research has indicated that miRNAs mainly bind to target mRNAs via the 3' untranslated region (3' UTR), but later studies have shown that some miRNAs can also bind to the 5' UTR or even the coding sequence (CDS) region 14, 15. MiRNAs that bind to the CDS region behave similarly to the classic negative regulation model through the 3' UTR. For example, Tay et al. found that multiple miRNAs can bind to the CDS region of Nanog, Oct4, and Sox2 transcripts and inhibit their protein expression 15.

In this study, we found that circRNA_103420 (cDCBLD2, hsa_circ_0066631) is critical for sorafenib resistance in HCC and further analyzed its function. Our data show that cDCBLD2 is upregulated in sorafenib-resistant hepatoma cells, and silencing cDCBLD2 could significantly increase sorafenib-induced apoptosis in HCC cells. Mechanistic studies suggested that cDCBLD2 can bind to miR-345-5p through a ceRNA mechanism, increase the stability of type IIA topoisomerase (TOP2A) mRNA as a miRNA sponge, and reduce the drug killing effect of sorafenib on HCC cells by inhibiting apoptosis. Different from the mechanism of most previously characterized miRNAs, miR-345-5p plays a negative regulatory role by binding to the TOP2A mRNA CDS region. Furthermore, silencing cDCBLD2 in a sorafenib-resistant mouse model could significantly boost the efficacy of sorafenib.

## Methods

### Cell lines and cell culture

For our study, we purchased three cell lines (SK-hep1, HepG2 and HCCLM3) from the American Type Culture Collection (ATCC, Manassas, VA, USA). Cell culture was conducted following the manufacturer's instructions. All cell lines were cultured in DMEM medium supplemented with 10% fetal bovine serum and maintained at standard incubation conditions of 37°C with 5% carbon dioxide. To establish sorafenib-resistant cell lines *in vitro*, we gradually increased the concentration of sorafenib over a period of 6 months. The resulting cell lines exhibited enhanced tolerance to higher concentrations of sorafenib treatment compared to their parental cell lines, and we defined them as a sorafenib-resistant cell lines. Subsequently, the sorafenib-resistant cells were maintained under continuous exposure to a concentration of 5μM sorafenib, which was chosen based on previous studies and its proven efficacy in exerting the desired pharmacological impact. For all subsequent *in vitro* experiments, the drug concentration of sorafenib used was 5 μM, and the duration of sorafenib treatment was set to 48-72 hours. This standardized approach allowed us to assess the effects of sorafenib consistently across different experiments.

### RNA Fluorescence *in situ* hybridization (FISH)

*In situ* hybridization of cDCBLD2 (CACATCCATCACCTTGTTTA+CY3/FAM), miRNA 345-5p (GAGCCCTGGACTAGGAGTCAGC+CY3) and 18s (genepharma, China) was performed using fluorescent-labeled specific probes which was from genepharma (China). The experiment was completed using RNA fluorescence *in situ* hybridization (FISH) kit (genepharma, China) according to the manufacturer's instructions. Images were obtained by Invitrogen™EVOS™ FL Auto 2 Imaging System (Thermo Fisher Scientific, Waltham, MA). The correlation of co localization fluorescence was analyzed using image J and Pearson test was performed.

### Nucleocytoplasmic separation

According to the manufacturer's instructions, PARIS™ Kit (Thermo Fisher Scientific, Waltham, MA) was employed for nucleocytoplasmic separation.

### Cell viability measurement

According to the manufacturer's instructions, cell growth inhibition was measured using the real-time cell analysis system (xCELLigence). Each pore initially contained approximately 5,000 cells, and the system automatically gathered cell growth data at 15-minute intervals.

The cell counting kit 8 (Yeasen, China) was used to detect cell viability. About 5,000 cells/well were incubated in 96-well plate. After treatment, the cells were incubated in working solution (10 µL CCK-8 assay solution and 100 µL DMEM) for approximately 1 hour. The absorption value was detected at a wavelength of 450 nm. The absence of cells in a pore was served as the blank control.

### Cell apoptosis assay

Following the specified treatment, all cells including floating cells and adherent cells were collected with trypsinization (0.25% Trypsin without EDTA, Gibco, China) and washed with PBS. According to the recommendations of the manufacturer, the apoptosis rate was measured using Annexin V-FITC/PI apoptosis kit (Multiscience, China) or Annexin V-Alexa Fluor647/PI Apoptosis Detection Kit (Yeasen, China). The cells were incubated with 5 µL Annexin V-FITC (or V-Alexa Fluor647) and 10 µL PI at room temperature, shielded from light, for 10 minutes. Flow cytometry was performed using BD LSRFortessa cell analyzer (BD Biosciences, USA). Finally, FlowJo software is used for data analysis.

### Clinical data analysis

The tissue samples were collected from 84 patients who exhibited sorafenib resistance, and the follow-up information was sourced from the General Surgery Department of Sir Run-Run Shaw Hospital. According to the level of TOP2A in tumor tissue detected by IHC method, the patients were divided into low expression group and high expression group. The log-rank test was used to analyze the correlation between TOP2A level and postoperative prognosis, OS and RFS rate of HCC patients.

### RNA pull down

Biotinylated-probe pull-down assays were performed in HCCLM3. The cells were lysed with a mixture of protein and IP buffer (Beyotime, China), along with protease inhibitor (Medchemexpress, USA), and RNase inhibitor (Sigma, USA). After prewash, the HCCLM3 cell lysates were incubated with MyOne™ Dynabead® Streptavidin M270 beads (Invitrogen, USA) at 4°C overnight. The 5' biotin-labeled cDCBLD2 probe (5'-biotin-gctaTCCACATCCATCACCTTGTTTATCTATA-3') or its control (5'-biotin-gctaTATAGATAAACAAGGTGATGGATGTGGA-3') was synthesized by Tsingke Biotech (Beijing, China) and incubated together. For miRNA biotinylated-probe pull-down assays, the 3' biotin-labeled miRNA345-5p probe (5'-GCUGACUCCUAGUCCAGGGCUC-3'-biotin) and its negative control were transfected into the cell in advance. Then the cells were lysed by the above method and lysates were incubated with MyOne™ Dynabead® Streptavidin C1 beads (Invitrogen, USA) at 4°C overnight. Collect the beads and wash several times, then use Trizol for RNA extraction.

### Dual-luciferase reporter assay

HCCLM3-SR cells were cultured and inoculated into 24-well plates. Next, the pmirGLO reporter vector containing either the wild-type or mutant sequences was transfected into the cells along with miR-345-5p mimics or NC (negative control) mimics. The transfection was carried out using Hieff Trans® Liposomal Transfection Reagent (Yeasen, China). The luminescence activity was detected with Dual Luciferase Reporter Gene Assay Kit (Yeasen, China) and chemiluminescence instrument.

### Immunohistochemical (IHC) staining

The tissue was fixed in 4% paraformaldehyde, followed by embedding in paraffin and cutting into 5 μm slice. After dewaxing, use Tris/EDTA buffer at pH 9.0 to boil for 5 minutes for antigen repair. Incubate the slide with 3% H_2_O_2_ for 30min to block the peroxidase, and then block it with 10% goat serum for 1h. Then it was incubated with the specific antibody of TOP2A (ab52934, 1:500, Abcam, Cambridge, MA) at 4℃ overnight. After washing with PBS, the slides were incubated with biotin-conjugated secondary antibody for 45 minutes and wash. IHC staining and DAB visualization of tissues were detected using GTVision III detection system (Gene Tech, China). Finally, the slides were stained with hematoxylin and then dehydrated. Two pathologists independently rated the staining intensity as "0" (negative), "1" (weak), "2" (moderate) or "3" (strong) by double-blind method. A score of 0 or 1 indicated low expression, and a score of 2 or 3 indicated high expression.

### Sorafenib-resistant HCC mouse model

Human tumor xenotransplantation model was employed to establish the subcutaneous cell-derived hepatocellular carcinoma (HCC) sorafenib-resistant mouse model. Tumor tissues obtained from HCC patients were fragmented and then implanted into the livers of NOD-SCID mice, creating the human tumor xenotransplantation model. After four weeks, the mice were administered 30 mg/kg/d of sorafenib via gavage to maintain the drug resistance of sorafenib. After 8 weeks of intragastric administration, the resulting tumors were cut into small pieces of equal volume (approximately 1mm^3^). These tissue fragments were then implanted into the armpits of 10 four-week-old BALB/c nude mice. After four weeks, mice with comparable and suitable tumor sizes were selected and randomly divided into two groups: the circRNA down-regulation group and the control group.

SiRNA transfection was employed to down-regulate circRNA. *In vivo* cholesterol coupled RIG-I siRNA (RiboBio, China) was used for siRNA transfection. Each tumor was locally injected with 5 nmol siRNA every 3 days for about 4 weeks. Throughout the experiment, all nude mice were given 30 mg/kg/d sorafenib by gavage. After the experiment, mice were sacrificed and tumor samples were collected for further study. The long axis (L) and short axis (W) of subcutaneous tumor were measured every 5 days. Tumor volume (mm^3^)=(L × W^2^)/2. All animal experiments were conducted in accordance with the guidelines reviewed by the Animal Ethics Committee of the Bioresources Center, the Science, Technology and Research Institution of the Sir Run-Run Shaw hospital, Zhejiang University School of Medicine.

### Statistical Analysis

Statistical analysis was conducted using GraphPad Prism 8. A T-test was employed to compare quantitative data between the two groups. The data were presented as the mean ± SEM (standard error of the mean) obtained from a minimum of three independent experiments. The two-tailed p-value of less than 0.05 was considered statistically significant.

Additional methods information can be found in Supplementary Materials.

## Results

### cDCBLD2 is highly expressed in sorafenib-resistant HCC cells

**Figure 1:**
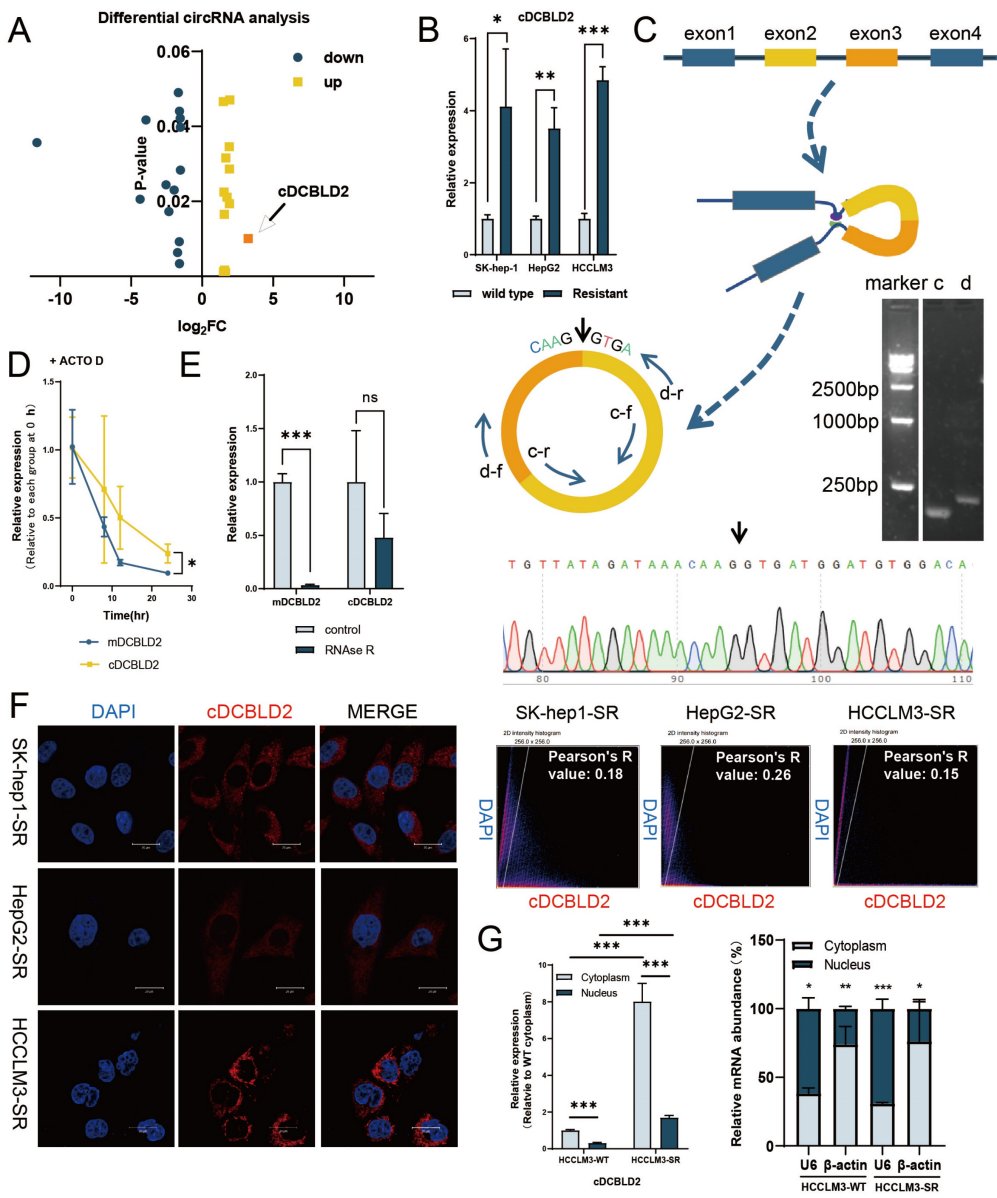
** cDCBLD2 is highly expressed in sorafenib-resistant HCC cells.** A. The circRNA microarray data showed that 14 circRNAs showed significantly increased expression levels in the drug resistant group. B. qPCR detection showed that cDCBLD2 expression levels were increased in SK-hep1-SR, HepG2-SR, and HCCLM3-SR cells. C. PCR and gel electrophoresis verified that cDCBLD2 is a cyclic RNA. D. Actinomycin D tests showed that cDCBLD2 was more stable than the linear gene. E. RNA enzyme R cleavage tests showed that cDCBLD2 was relatively stable. F. FISH analysis suggested that cDCBLD2 was mainly located in the cytoplasm. Pearson's test suggested a low correlation between cDCBLD2 and DAPI signal. G. Nucleo-cytoplasmic separation tests showed that cDCBLD2 was mainly located in the cytoplasm. qPCR data for identifying the efficiency of nuclear cytoplasmic separation.

Accumulating evidence indicates that circRNAs are involved in various cancer-related processes. To identify circRNAs that may be involved in mediating sorafenib resistance, we successfully constructed three sorafenib-resistant HCC cell lines, namely SK-Hep1-SR, HepG2-SR, and HCCLM3-SR, adopting the long-term treatment method of slowly increasing the drug concentration. It was also verified that these three cell lines and their parent cell lines had different tolerance levels to sorafenib **(Fig. S1A)**. To further reveal the potential mechanism of sorafenib resistance in HCC, our group previously screened the circRNAs expressed in the constructed sorafenib-resistant cell line (HepG2-SR) and wild-type cell line (HepG2-WT) using a circRNA array 9. We observed 28 circRNAs (*P*<0.05) that were significantly differentially expressed between the drug resistant and non-drug resistant groups, of which 14 were significantly increased in the drug resistant group. Among the 14 upregulated circRNAs, circRNA_103420 (cDCBLD2, hsa_circ_0066631) had the highest fold change and higher expression levels compared with the previously discovered circRNA-SORE, which could lead to increased sorafenib resistance among HCC cells through exosomes 9 **(Fig. 1A, S1B)**. Further analysis using qPCR indicated that cDCBLD2 expression levels were increased in SK-Hep1-SR, HepG2-SR, and HCCLM3-SR cells relative to their corresponding WT cell line **(Fig. 1B)**, suggesting that this circRNA may play an important role in HCC resistance to sorafenib.

To determine the origin of cDCBLD2, we queried the UCSC genome browser and found that cDCBLD2 is generated from reverse splicing of exons 2 and 3 of the DCBLD2 (discoidin, CUB and LCCL domain containing 2) gene. Next, we designed convergent and divergent primers, then used PCR and gel electrophoresis to verify that cDCBLD2 is a cyclic RNA** (Fig. 1C)**. Actinomycin D and RNase R cleavage tests in HCCLM3 cells suggested that cDCBLD2 was more stable than the linear transcript, further demonstrating that cDCBLD2 is a circRNA **(Fig. 1D, E)**. FISH assays were used to verify that cDCBLD2 is mainly localized to the cytoplasm **(Fig. 1F)**. These data were corroborated by nucleocytoplasmic separation tests. These results also suggested that cDCBLD2 levels are significantly increased in the drug-resistant cells **(Fig. 1G)**.

### cDCBLD2 can promote proliferation and suppress apoptosis in HCC cells

**Figure 2:**
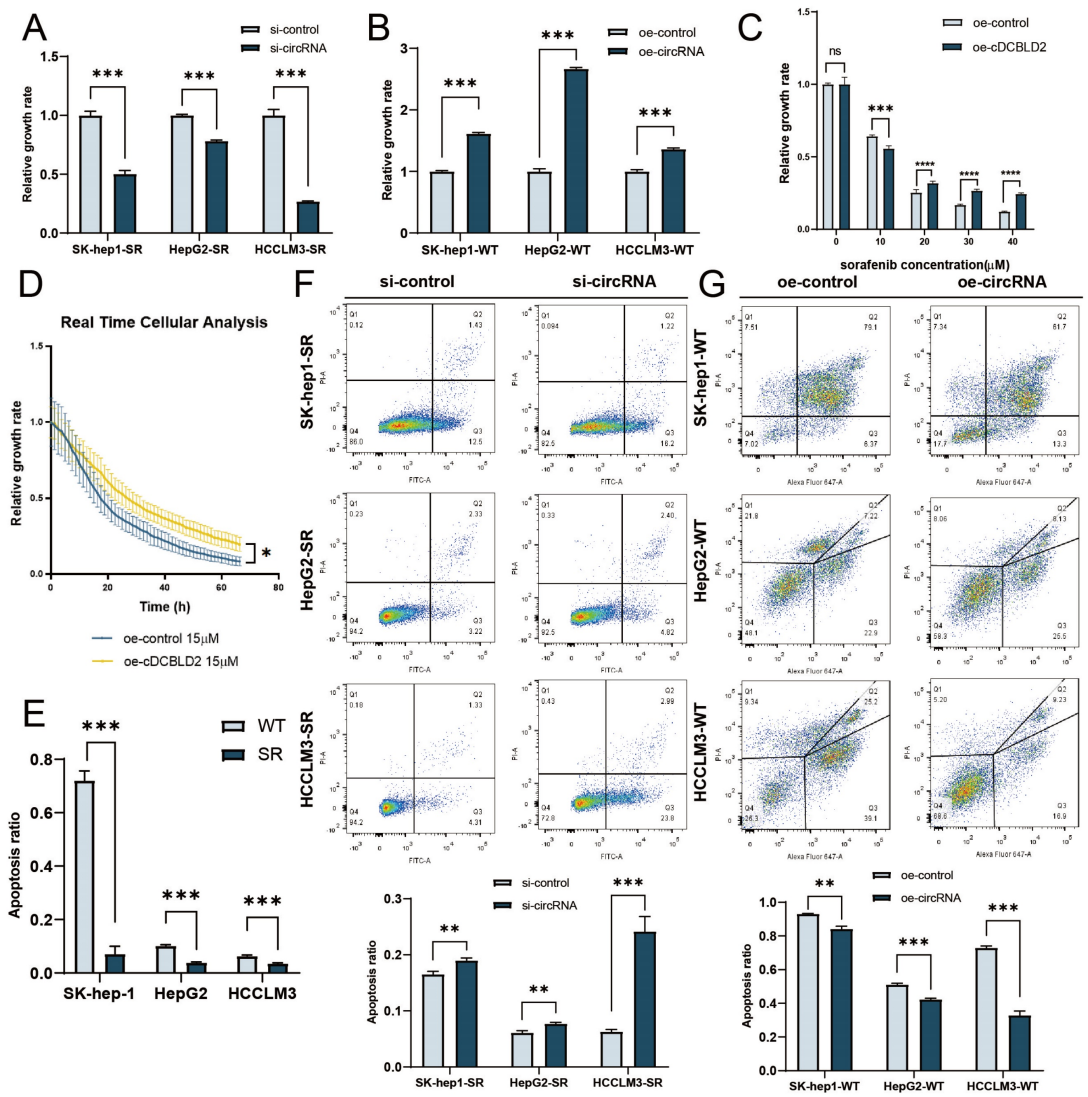
** cDCBLD2 can promote proliferation and suppress apoptosis in HCC cells.** A. Using an siRNA to knock down cDCBLD2 expression in SK-hep1-SR, HepG2-SR, and HCCLM3-SR cells can significantly reduce the viability of cells under sorafenib treatment. B. Overexpression of cDCBLD2 in SK-hep-1, HepG2, and HCCLM3 cells can increase the viability of cells under sorafenib treatment. C. CCK8 tests showed that overexpression of cDCBLD2 increased the tolerance of HCCLM3-WT cells to sorafenib treatment. D. Real-time unmarked cell analysis showed that overexpression of cDCBLD2 in HCCLM3-WT cells could significantly reduce the inhibition of cells treated with sorafenib. E. With sorafenib treatment, the percentage of apoptotic cells of three sorafenib-resistant cell lines was significantly lower than that of their parent cell lines. F. With sorafenib treatment, cDCBLD2 knockdown in SK-Hep1-SR, HepG2-SR, and HCCLM3-SR cells led to increased rates of apoptosis. G. Overexpression of cDCBLD2 in three wild-type cell lines resulted in decreased apoptosis rates.

Previous work demonstrated that three sorafenib-resistant cell lines treated with sorafenib exhibited faster growth rates compared with their parent cell lines 9. To explore the role of cDCBLD2 in sorafenib resistance, we used siRNA transfection and plasmid overexpression methods to reduce or increase the cDCBLD2 levels in sorafenib-resistant cells or WT cells, respectively. To knock down cDCBLD2 expression, we designed two siRNA sequences (si-cDCBLD2 1 and si-cDCBLD2 2). Subsequently, we used siRNA transfection and qRT-PCR experiments to examine the efficiency of cDCBLD2 knockdown in three sorafenib-resistant cell lines, finding that si-cDCBLD2 1 had a higher efficiency value **(Fig. S1C)**. Therefore, we chose this siRNA to use in subsequent experiments. We also verified the knockdown efficiency of si-cDCBLD2 1 under sorafenib treatment, also observing an excellent knockdown effect **(Fig. S1D)**. Simultaneously, we also verified the overexpression efficiency of cDCBLD2 in wild-type cells after transient plasmid transfection, and obtained promising results **(Fig. S1E)**. We then examined if these altered cDCBLD2 levels had any effect on cell response to sorafenib. The results suggested that knocking down cDCBLD2 could significantly enhance the killing effect of sorafenib on drug-resistant cells, which was manifested by a decrease in cell viability **(Fig. 2A)**. Overexpressing cDCBLD2 had the opposite effect **(Fig. 2B)**. CCK8 assays also indicated that higher levels of cDCBLD2 could support the tolerance of cells to increased sorafenib concentrations **(Fig. 2C)**. Real-time label-free cell activity analysis suggests that overexpression of cDCBLD2 can reduce the inhibition of sorafenib on cell growth **(Fig. 2D)**.

We further verified the apoptosis rates of three sorafenib-resistant cell lines, finding that the percentage of apoptotic cells was significantly lower than that of the corresponding parent cell line following sorafenib treatment **(Fig. 2E)**. However, following cDCBLD2 knockdown in SK-Hep1-SR, HepG2-SR, and HCCLM3-SR cells treated with sorafenib, the proportion of apoptotic cells increased** (Fig. 2F)**. Overexpression of cDCBLD2 in the three WT cell lines resulted in decreased apoptosis rates **(Fig. 2G).** In summary, these data demonstrate that cDCBLD2 can promote HCC cell proliferation with sorafenib treatment, but inhibits their apoptosis.

### cDCBLD2 affects sorafenib resistance in HCC cells by regulating TOP2A

**Figure 3:**
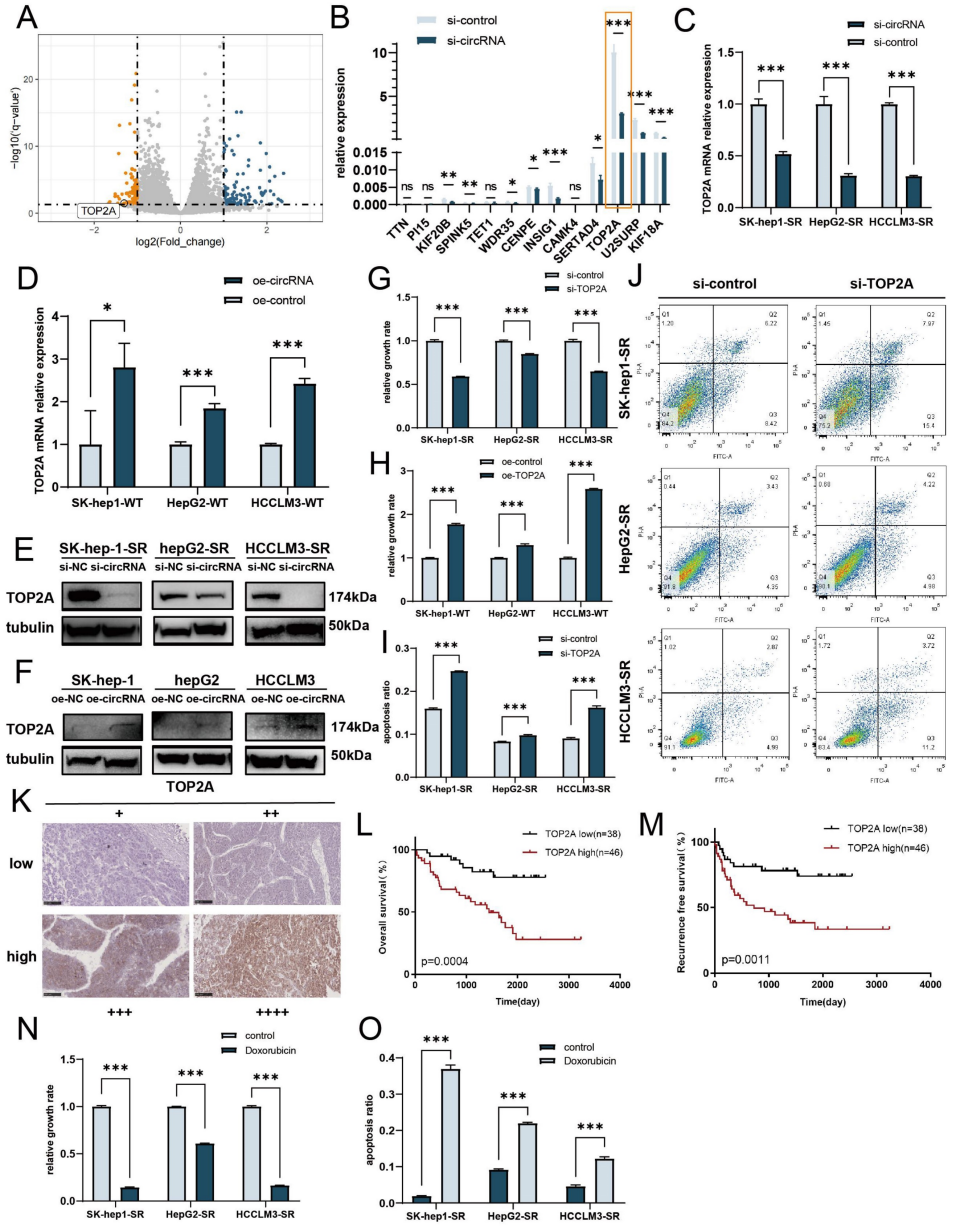
** cDCBLD2 affects sorafenib resistance in HCC cells by regulating TOP2A expression.** A. Volcano plots of the differentially expressed genes between HCCLM3-SR samples, with or without cDCBLD2 knockdown, by PolyA sequencing. B. qPCR results of downregulated genes in HCCLM3-SR cells after knocking down cDCBLD2. C. With sorafenib treatment, TOP2A mRNA levels significantly decreased in SK-Hep1-SR, HepG2-SR, and HCCLM3-SR cells with cDCBLD2 knockdown. D. TOP2A mRNA levels after overexpression of cDCBLD2 in SK-Hep1-WT, HepG2-WT, and HCCLM3-WT cells. E,F. Western blot analysis of sorafenib-resistant cells with cDCBLD2 knockdown or wild-type cells with cDCBLD2 overexpression. G,H. CCK8 assays of knocking down or overexpressing TOP2A with sorafenib treatment. I,J. Apoptosis levels with knocking down or overexpressing TOP2A and sorafenib treatment. K. Representative IHC images of TOP2A protein expression in HCC clinical samples (20×). The patients were divided into two groups. The low expression group includes patients with negative and weak TOP2A protein expression, while the high expression group includes patients with moderate and strong TOP2A protein expression. L,M. The log-rank test was used to generate survival curves of the overall survival and relapse-free survival rates. N,O. CCK8 and apoptosis tests with or without doxorubicin treatment.

Because cDCBLD2 originates from the DCBLD2 gene, we first tested whether this circRNA can regulate its host gene. We detected the mRNA and protein levels of DCBLD2 after cDCBLD2 knockdown, but did not find any obvious consistent changes in the three sorafenib-resistant HCC cell lines **(Fig. S1F, G)**. To further study the downstream mechanism of cDCBLD2 in sorafenib resistance, we performed PolyA-Seq analysis on RNA samples from HCCLM3-SR cells with cDCBLD2 knockdown and control cells. We used the DESeq2 method to analyze the differential expression of known mRNAs, identifying 117 upregulated mRNAs and 97 downregulated mRNAs (| log2 (FoldChange) | > 1, Q value < 0.05) **(Fig. 3A).** The top 20 most downregulated mRNAs in the cDCBLD2 knockdown group were analyzed. Thirteen of these could encode protein and were selected for qPCR analysis in HCCLM3-SR cells. Interestingly, TOP2A mRNA levels were significantly higher than those of the other 12 mRNAs, but were significantly lower than that of the control group after cDCBLD2 knockdown **(Fig. 3B)**. We further reduced or increased cDCBLD2 levels in sorafenib-resistant cells or WT cells, respectively. The results showed that when SK-Hep1-SR, HepG2-SR, and HCCLM3-SR cells were treated with cDCBLD2 siRNA and sorafenib, TOP2A mRNA levels significantly decreased **(Fig. 3C).** However, after cDCBLD2 overexpression in SK-Hep1-WT, HepG2-WT, and HCCLM3-WT cells, TOP2A mRNA levels increased significantly** (Fig. 3D)**. Collectively, these experiments suggest that TOP2A mRNA may be a downstream target of cDCBLD2.

We next verified TOP2A protein expression in these cells. With both sorafenib treatment and cDCBLD2 knockdown in SK-Hep1-SR, HepG2-SR, and HCCLM3-SR cells, TOP2A protein levels were also significantly decreased **(Fig. 3E, S2A)**, while they were increased following cDCBLD2 overexpression in the WT cell lines **(Fig. 3F, S2B)**. In addition, with sorafenib treatment, we knocked down TOP2A expression in the drug-resistant cells and observed decreased cell growth rates **(Fig. 3G)**. However, overexpression of TOP2A in the WT cells promoted cell growth **(Fig. 3H).** Correspondingly, there was a significantly higher proportion of apoptotic drug-resistant cells following TOP2A knockdown compared with the control group **(Fig. 3I, J)**. When TOP2A was overexpressed in wild-type cells, there was a significant reduction in apoptosis levels in cells treated with sorafenib **(Fig. S2C)**.

To verify the role of TOP2A in clinical sorafenib-resistant HCC patients, 84 HCC patients with a regular medication history of sorafenib and complete prognosis information were selected. The clinical features of these patients are presented in **Table 1**. Immunohistochemistry was performed to evaluate TOP2A protein levels in tumor tissues, and patients were divided into two groups according to their expression of TOP2A **(Fig. 3K)**. Analysis of the clinicopathological information indicated that the TOP2A expression level is related to the degree of tumor differentiation, recurrence, and metastasis of HCC patients treated with sorafenib** (Table 1)**. Survival analysis showed that patients with higher expression of TOP2A had significantly worse overall survival (OS) **(Fig. 3L, *P*=0.0004)** and relapse-free survival (RFS) **(Fig. 3M, *P*=0.0011)**. These results suggested that HCC patients with high TOP2A expression levels had worse efficacy for sorafenib, meaning that they had more resistance to sorafenib. Overall, these clinical data further demonstrate the role of cDCBLD2 in promoting HCC tumor progression and sorafenib resistance through TOP2A. Fortunately, a commonly used anti-tumor drug, doxorubicin, can selectively inhibit TOP2A in clinical practice. We found that doxorubicin treatment could significantly enhance the killing effect of sorafenib in drug-resistant cells, reducing its activity **(Fig. 3N)** and increasing apoptosis rates **(Fig. 3O, S1H)**. However, the combination of doxorubicin and sorafenib is the limitation of this study, as further more clinical trials are needed to confirm its feasibility.

### cDCBLD2 levels are negatively correlated with miR-345-5p expression levels

**Figure 4:**
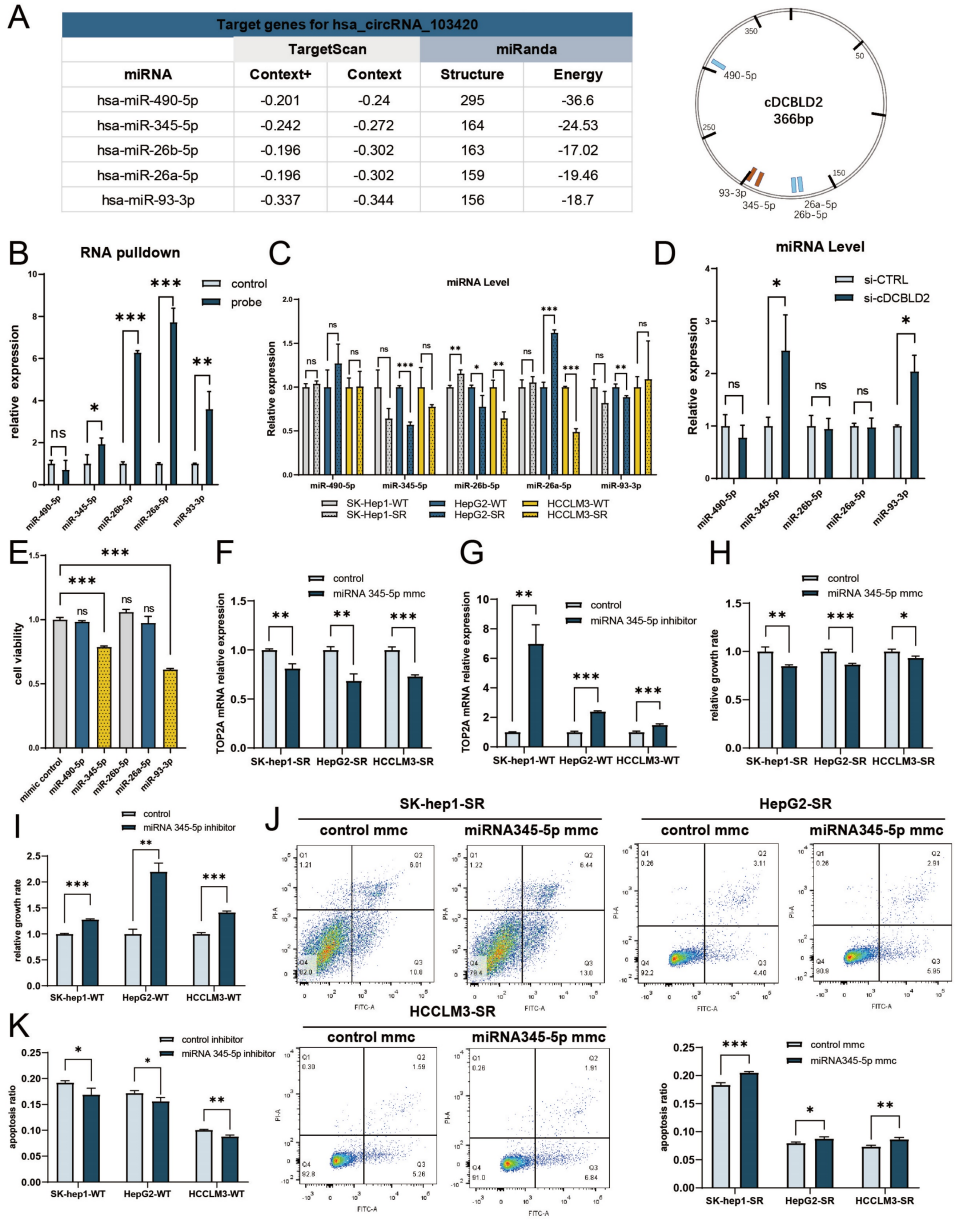
** cDCBLD2 levels are negatively correlated with miR-345-5p expression levels.** A. miRanda was used to predict that there are possible miRNA binding sites in the cDCBLD2 sequence. B. RNA pulldown tests were used to detect the miRNAs binding to cDCBLD2 in HepG2-SR cells. C. Corresponding miRNA expression levels in sorafenib-resistant and wild-type cells. D. Intracellular miRNA levels after knocking down cDCBLD2 in HepG2-SR cells. E. The effect of overexpression of corresponding miRNAs on cell viability was detected in HepG2-SR cells. F. MiRNA mimic overexpression tests showed that increasing miR-345-5p levels with sorafenib treatment generally reduced TOP2A mRNA levels in HCC sorafenib-resistant cells. G. With sorafenib treatment, inhibiting miR-345-5p significantly increased TOP2A mRNA levels in wild-type HCC cells. H. Increasing miR-345-5p can significantly reduce the activity of sorafenib-resistant cells with sorafenib treatment. I. Under the action of sorafenib, inhibiting miR-345-5p showed enhanced wild-type cell activity. J. Higher miR-345-5p levels led to increased sorafenib-resistant cell apoptosis rates. K. Inhibition of miR-345-5p resulted in decreased apoptosis rates in wild-type cells.

When investigating the molecular mechanism by which cDCBLD2 affects TOP2A mRNA levels, we first considered the common miRNA sponge role of circRNAs. We used bioinformatics tools to predict the possible miRNA targets of cDCBLD2, including the miRanda miRNA target gene prediction software. According to the scores, we selected the top five potential miRNA targets of cDCBLD2: miR-490-5p, miR-345-5p, miR-26b-5p, miR-26a-5p, and miR-93-3p **(Fig. 4A)**. Using RNA pulldown and other experiments, the data suggested that miR-345-5p, miR-26b-5p, miR-26a-5p, and miR-93-3p potentially competitively bind to cDCBLD2 **(Fig. 4B)**. We also evaluated the expression levels of these miRNAs in sorafenib-resistant cells and WT cells, finding that miR-345-5p was generally decreased in the three drug-resistant cell lines **(Fig. 4C)**. Using siRNA technology to knock down cDCBLD2 expression, miR-345-5p and miR-93-3p levels both increased, suggesting that these two miRNAs may be regulated by cDCBLD2 **(Fig. 4D)**. We hypothesized that cDCBLD2 can inhibit the biological functions of these miRNAs by binding them via a ceRNA mechanism. Then, we examined the effect of increasing the levels of these miRNAs on sorafenib efficacy through miRNA mimic overexpression. The results suggested that miR-345-5p and miR-93-3p overexpression each enhanced the killing effect of sorafenib on cells** (Fig. 4E)**, suggesting that miR-345-5p and miR-93-3p are involved in regulating cell response to sorafenib. Through various experiments, our data indicate that cDCBLD2 can participate in regulating cell response to sorafenib through miR-345-5p or miR-93-3p.

To further determine if cDCBLD2 regulates TOP2A mRNA levels through miR-345-5p or miR-93-3p, we verified the relationship between these miRNAs and TOP2A mRNA. Following sorafenib treatment and miRNA mimic overexpression in sorafenib-resistant cells, we found that higher miR-345-5p levels generally reduced TOP2A mRNA levels** (Fig. 4F)**. However, no consistent trend was observed for TOP2A mRNA expression after miR-93-3p overexpression **(Fig. S1I)**. Combined with previous experiments, these results suggested that cDCBLD2 mainly works via miR-345-5p. Using a specific miR-345-5p inhibitor, we saw significantly increased TOP2A mRNA levels in WT cells** (Fig. 4G)**. These results suggest that TOP2A mRNA expression is negatively regulated by miR-345-5p.

We used miRNA mimic overexpression and miRNA inhibitor experiments to increase or decrease miR-345-5p levels in sorafenib-resistant cells or WT cells, respectively, then examined the effects of these changes on the response to sorafenib. The results suggested that overexpressing miR-345-5p could significantly enhance the killing effect of sorafenib on sorafenib-resistant cells, as seen by decreased cell activity **(Fig. 4H)** and increased apoptosis rates** (Fig. 4J)**. Inhibiting miR-345-5p in WT cells had opposite effects **(Fig. 4I, K, S2D)**. These data show that cDCBLD2 expression patterns are negatively correlated with miR-345-5p levels.

### cDCBLD2 can stabilize TOP2A mRNA by competitively binding miR-345-5p, which directly binds to the CDS region of TOP2A

**Figure 5:**
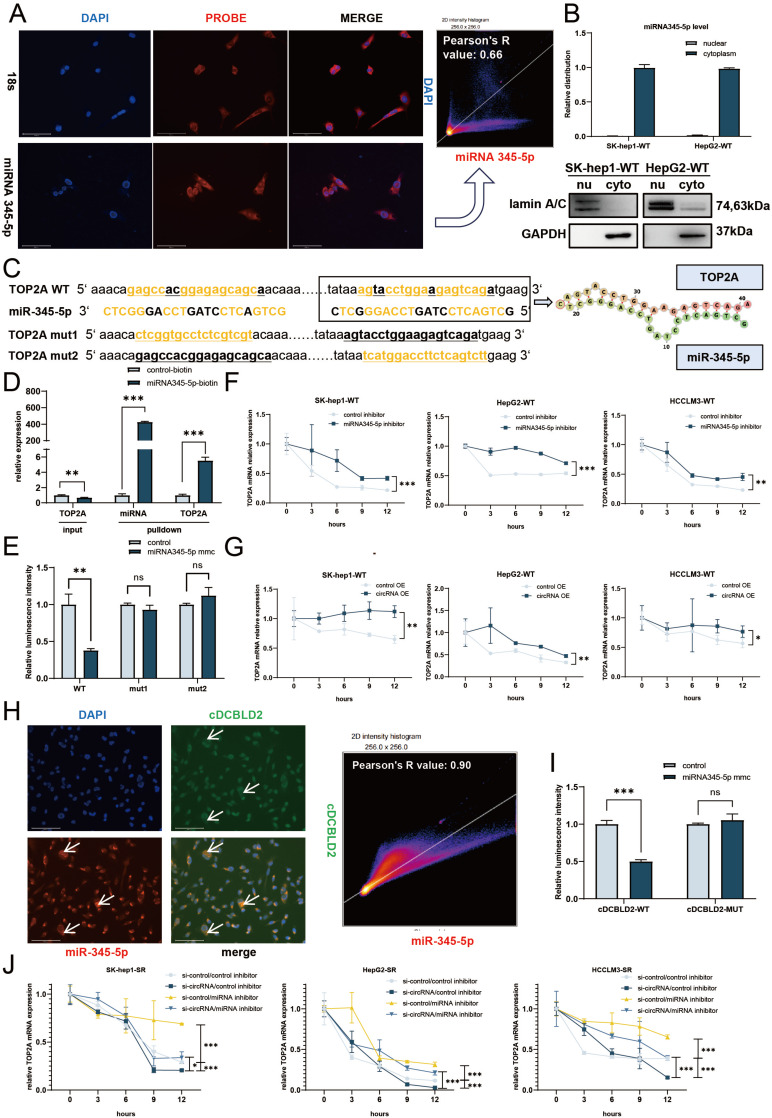
** cDCBLD2 can stabilize TOP2A mRNA by competitively binding miR-345-5p, which directly binds to the CDS region of TOP2A.** A. FISH tests in SK-hep1-WT cells showed that miR-345-5p was expressed more in the cytoplasm. Pearson's tests suggested a moderate correlation between miR-345-5p and DAPI. B. miR-345-5p was mainly localized in the cytoplasm according to the nucleocytoplasmic separation test. C. The miR-345-5p binding sites in TOP2A mRNA, as predicted by the database. D. RNA pulldown was performed with a miR-345-5p-biotin probe, and TOP2A mRNA was enriched. E. Dual luciferase reporter assays co-transfected with miR-345-5p mimic or its control using TOP2A wild-type plasmids (TOP2A-WT) containing potential miR-345-5p binding sites and plasmids with binding site mutations (TOP2A-MUT1, TOP2A-MUT2). F. With sorafenib treatment, TOP2A mRNA stability was increased in three wild-type HCC cell lines after inhibition of miR-345-5p levels by actinomycin D treatment. G. After overexpression of cDCBLD2 in three HCC wild-type cell lines, TOP2A mRNA stability increased under actinomycin D treatment. H. FISH co-localization of cDCBLD2 and miR-345-5p. Pearson's tests suggested a high correlation. I. Dual luciferase reporter experiments on miR-345-5p binding sites and cDCBLD2. J. Rescue experiment of TOP2A stability under actinomycin D treatment.

To better understand the molecular mechanism of miR-345-5p, we first investigated the localization of miR-345-5p in cells using FISH and nucleocytoplasmic separation experiments. The results showed that miR-345-5p was expressed more in the cytoplasm **(Fig. 5A, B)**. Therefore, we speculated that miR-345-5p mainly played its role in the cytoplasm. Next, we used the bioinformatics tool miRWalk (http://129.206.7.150/). Two potential miR-345-5p binding sites were found in TOP2A mRNA **(Fig. 5C)**. Interestingly, these binding sites were both located in the CDS region. RNA pulldown and dual-luciferase reporter gene experiments confirmed this hypothesis. RNA pulldown was performed after transfecting HCCLM3-SR cells with a biotin-labeled miR-345-5p probe or control probe. TOP2A mRNA was preferentially enriched in the miR-345-5p-biotin probe group, confirming that miR-345-5p and TOP2A mRNA could directly interact **(Fig. 5D)**. We then used a TOP2A WT plasmid (TOP2A-WT) containing potential miR-345-5p binding sites and a mutant plasmid (TOP2A-MUT1, TOP2A-MUT2) with mutated versions of the two predicted binding sites. These plasmids were co-transfected into HCCLM3-SR cells with miR-NC or miR-345-5p mimic. The results showed that the relative luciferase activity of the miR-345-5p mimic group transfected with TOP2A-WT plasmid was significantly lower than that of the negative control group. However, the relative luciferase activity of the miR-345-5p mimic group transfected with TOP2A-MUT1 and TOP2A-MUT2 plasmid did not significantly differ from the negative control group **(Fig. 5E)**. These findings suggest that miR-345-5p can directly regulate TOP2A mRNA through these two binding sites.

From these data, we speculated that miR-345-5p plays a role in the cytoplasm after binding to TOP2A mRNA. However, the two miR-345-5p binding sites are located in the TOP2A mRNA CDS region, which is relatively rare. Because of this, we examined TOP2A mRNA stability under different treatment conditions to further verify if its mechanism of action is the same as classical miRNA-mediated negative regulation via the 3' UTR 15. We found that with sorafenib treatment, TOP2A mRNA stability increased in the three wild-type HCC cell lines after miR-345-5p inhibition and actinomycin D treatment **(Fig. 5F)**. These data demonstrate that miR-345-5p can directly interact with TOP2A mRNA via binding sites in its CDS region, which negatively affects its expression levels by reducing mRNA stability.

After further verification using cDCBLD2 overexpression in these cell lines, a similar trend was observed with increased stability of TOP2A mRNA **(Fig. 5G)**. We then conducted FISH co-localization experiments on cDCBLD2 and miR-345-5p, finding that they exhibited spatial consistency **(Fig. 5H)**. This suggested that these molecules possibly influence each other's functionality. Next, we performed a sequence alignment of cDCBLD2 and miR-345-5p and identified their potential binding sites **(Fig. S2E)**. The cDCBLD2 wild-type plasmid (cDCBLD2-WT-pmirGLO) and mutant plasmid (cDCBLD2-MUT-pmirGLO) with the predicted miR-345-5p binding site mutated were co-transfected with miR-NC or miR-345-5p mimic. The results showed that in the HCCLM3-SR cell line, the relative luciferase activity of the miR-345-5p mimic group transfected with the cDCBLD2-WT plasmid was significantly lower than that of the control group. However, the miR-345-5p mimic group transfected with the cDCBLD2-MUT plasmid showed no significant decrease in relative luciferase activity compared with the control group **(Fig. 5I)**. This suggests that this site is indeed a binding site between cDCBLD2 and miR-345-5p. Moreover, we found that inhibition of miR-345-5p could rescue the decreased stability of TOP2A mRNA after actinomycin D treatment because of the reduction of cDCBLD2 **(Fig. 5J)**. In summary, miR-345-5p can promote the degradation of TOP2A mRNA by binding to its CDS region. Additionally, cDCBLD2 inhibits the function of TOP2A by competitively binding to miR-345-5p, thereby stabilizing TOP2A mRNA.

### cDCBLD2 can stabilize TOP2A protein through a miR-345-5p sponge mechanism and affect caspase-3-mediated apoptosis

**Figure 6:**
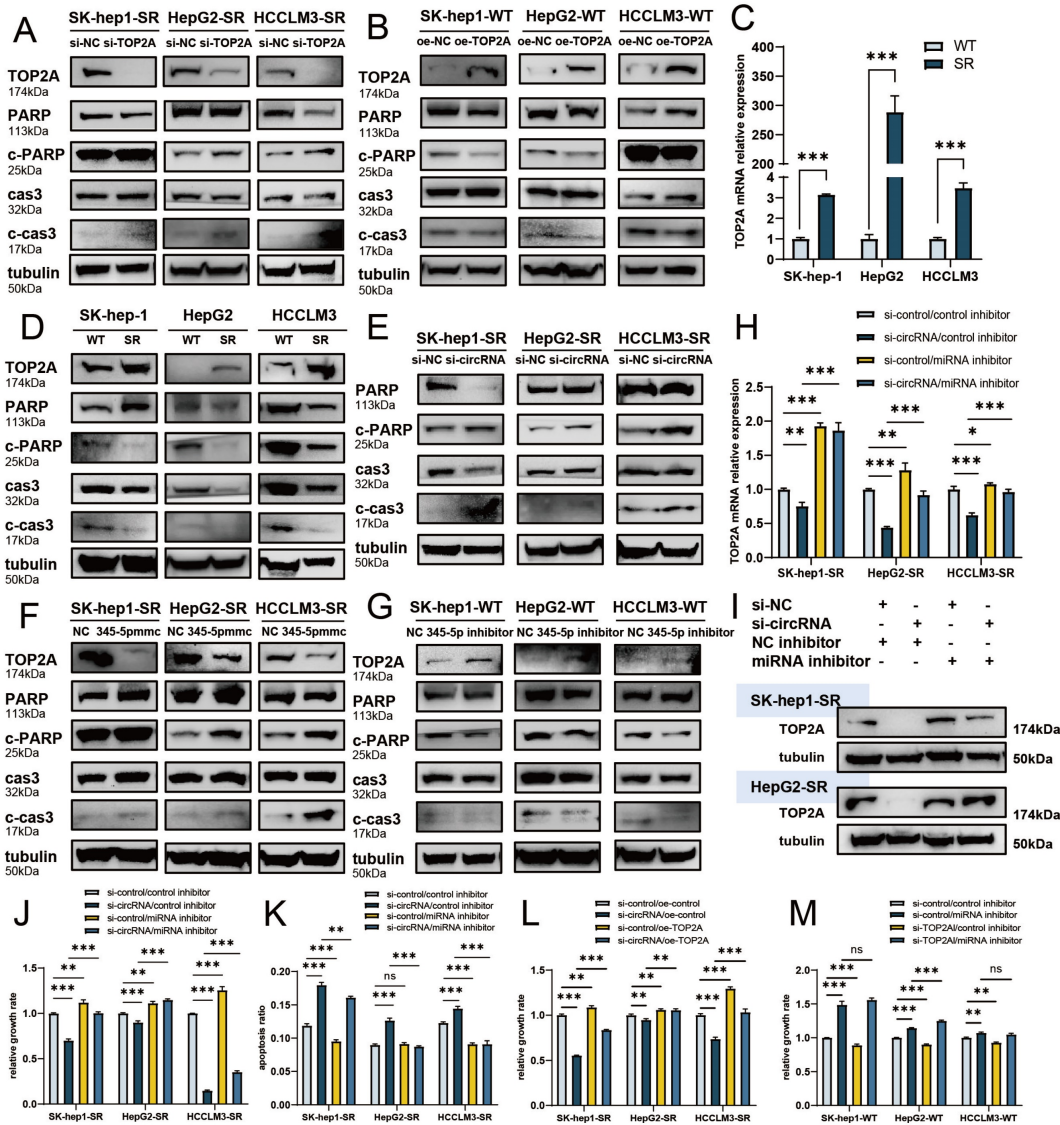
** cDCBLD2 can stabilize TOP2A protein through a miR-345-5p sponge mechanism and affect caspase-3-mediated apoptosis.** A,B. Western blot analysis was used to detect relevant protein levels following knockdown or overexpression of TOP2A in wild-type HCC cells and sorafenib-resistant cells treated with sorafenib. C. In the three pairs of SK-Hep-1, HepG2, and HCCLM3 cells treated with sorafenib, the TOP2A mRNA levels were significantly higher in sorafenib-resistant cells compared with wild-type cells. D. Western blot analysis was used to detect relevant protein levels in wild-type and sorafenib-resistant cells treated with sorafenib. E. Western blot analysis was used to detect relevant protein levels following knockdown of cDCBLD2 in sorafenib-resistant cells treated with sorafenib. F,G. Western blot analysis was used to detect relevant protein levels following overexpression of miR-345-5p in sorafenib-resistant cells or inhibition of miR-345-5p in wild-type HCC cells treated with sorafenib. H. qRT-PCR was used to detect TOP2A mRNA levels with or without cDCBLD2 knockdown or miR-345-5p inhibition in sorafenib-resistant cells treated with sorafenib. I. Western blot analysis was used to detect TOP2A protein levels in SK-Hep1-SR and HepG2-SR cells with or without cDCBLD2 knockdown or miR-345-5p inhibition with sorafenib treatment. J. CCK-8 assays were used to detect the activity of drug-resistant cells treated with sorafenib with various combinations of cDCBLD2 knockdown and miR-345-5p inhibition. K. Flow cytometry was used to detect apoptosis rates in drug-resistant cells treated with sorafenib with or without cDCBLD2 knockdown or miR-345-5p inhibition. L. CCK-8 assays were used to detect the activity of drug-resistant cells treated with sorafenib with various combinations of cDCBLD2 knockdown and TOP2A overexpression. M. CCK-8 assays were used to detect the activity of wild-type cells treated with sorafenib with various combinations of miR-345-5p inhibition and TOP2A knockdown.

In previous experiments, we found that cell apoptosis rates changed following sorafenib treatment. Most factors that can trigger apoptosis ultimately work through the signal transduction pathway mediated by caspase-3. Previous studies have also reported that TOP2A can be characterized by poly ADP-ribose polymerase-1 (PARP-1) cleavage and caspase-3 activation, thus triggering the apoptosis pathway 16,17. Caspase-3 can promote apoptosis by degrading its enzyme substrate, such as PARP 18. We found that with sorafenib treatment, the protein expression levels of apoptosis-indicating proteins cleaved-caspase-3 (c-cas3) and cleaved-PARP (c-PARP) were significantly higher following knockdown of TOP2A in sorafenib-resistant cells **(Fig. 6A, S3A-C),** suggesting that capsase-3-mediated apoptosis was promoted. Overexpression of TOP2A in WT cells had opposite results** (Fig. 6B, S3D-F)**.

We also verified TOP2A expression levels in SK-Hep1, HepG2, and HCCLM3 WT and sorafenib-resistant HCC cells with sorafenib treatment. Both TOP2A mRNA **(Fig. 6C)** and protein **(Fig. 6D, S3G)** levels were significantly higher in sorafenib-resistant cells than in WT cells. Therefore, TOP2A may indeed be a functional protein that plays a role in sorafenib resistance. We also found that the protein expression levels of c-cas3 and c-PARP were significantly lower in drug-resistant cells than in WT cells **(Fig. 6D, S3H, I)**, further verifying that the caspase-3-mediated apoptosis pathway is related to sorafenib resistance.

In previous experiments, we found that TOP2A protein levels were decreased after cDCBLD2 knockdown in SK-Hep1-SR, HepG2-SR, and HCCLM3-SR cells treated with sorafenib. Interestingly, the expression levels of apoptotic indicator proteins c-cas3 and c-PARP were increased **(Fig. 6E, S3J, K)**. We also examined the protein levels of TOP2A, c-cas3, and c-PARP following miR-345-5p mimic overexpression in drug-resistant cells, finding that the protein levels of TOP2A decreased, while those of c-cas3 and c-PARP increased **(Fig. 6F, S3L-N)**. This was consistent with our expected results. Additionally, after inhibiting miR-345-5p in WT SK-Hep1, HepG2, and HCCLM3 cells, the opposite results were obtained **(Fig. 6G, S3O-Q)**.

In our study, knockdown of cDCBLD2 could reduce TOP2A mRNA levels in drug-resistant cells treated with sorafenib, which partially promoted cell death induced by sorafenib. However, inhibiting miR-345-5p rescued the observed decrease of TOP2A mRNA that resulted from cDCBLD2 knockdown **(Fig. 6H)**. TOP2A protein levels were verified in SK-Hep1-SR and HepG2-SR cells. Inhibiting miR-345-5p could also rescue the reduction in TOP2A protein levels caused by cDCBLD2 knockdown **(Fig. 6I, S4A)**.

**Figure 7:**
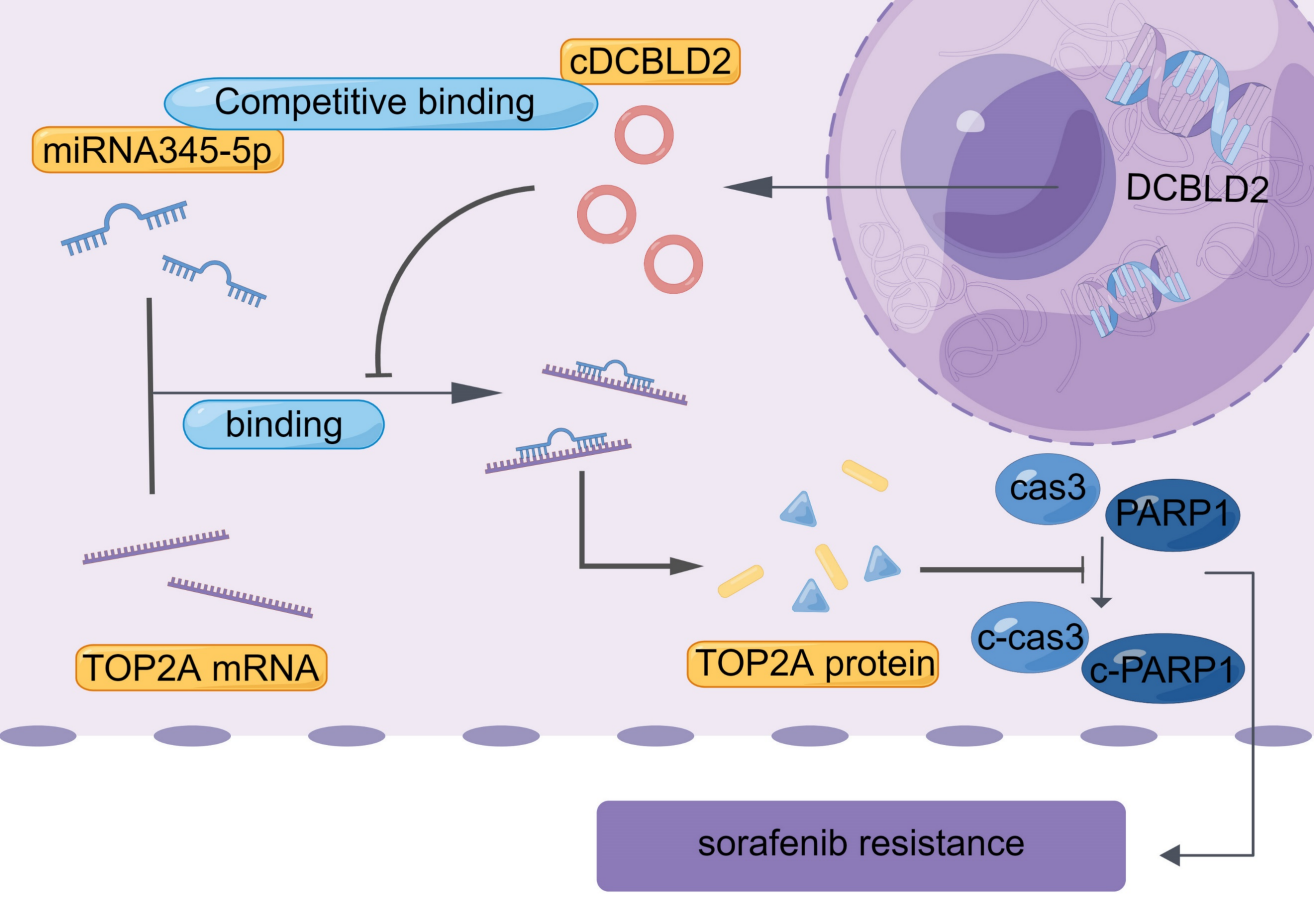
Proposed mechanistic depiction of sorafenib resistance. This was generated using figdraw.

We also examined cell growth and apoptosis rates. With sorafenib treatment, knocking down cDCBLD2 can inhibit drug-resistant HCC cell growth, but this could be partially rescued when miR-345-5p was inhibited **(Fig. 6J)**. Similarly, inhibiting miR-345-5p could also rescue the increased apoptosis rates resulting from cDCBLD2 knockdown **(Fig. 6K, S4B)**. Furthermore, we verified that overexpression of TOP2A could rescue the increased killing of sorafenib caused by knocking down cDCBLD2 **(Fig. 6L)**. Additionally, after inhibiting miR-345-5p and then knocking down TOP2A expression, the cell killing effect of sorafenib could not be enhanced **(Fig. 6M)**. These data further confirm that cDCBLD2 can competitively regulate TOP2A expression through a miR-345-5p sponge mechanism, thus affecting both sorafenib resistance and the caspase-3-mediated apoptosis process in HCC cells **(Fig. 7)**.

### Knockdown of cDCBLD2 can inhibit HCC sorafenib resistance *in vivo*

**Figure 8:**
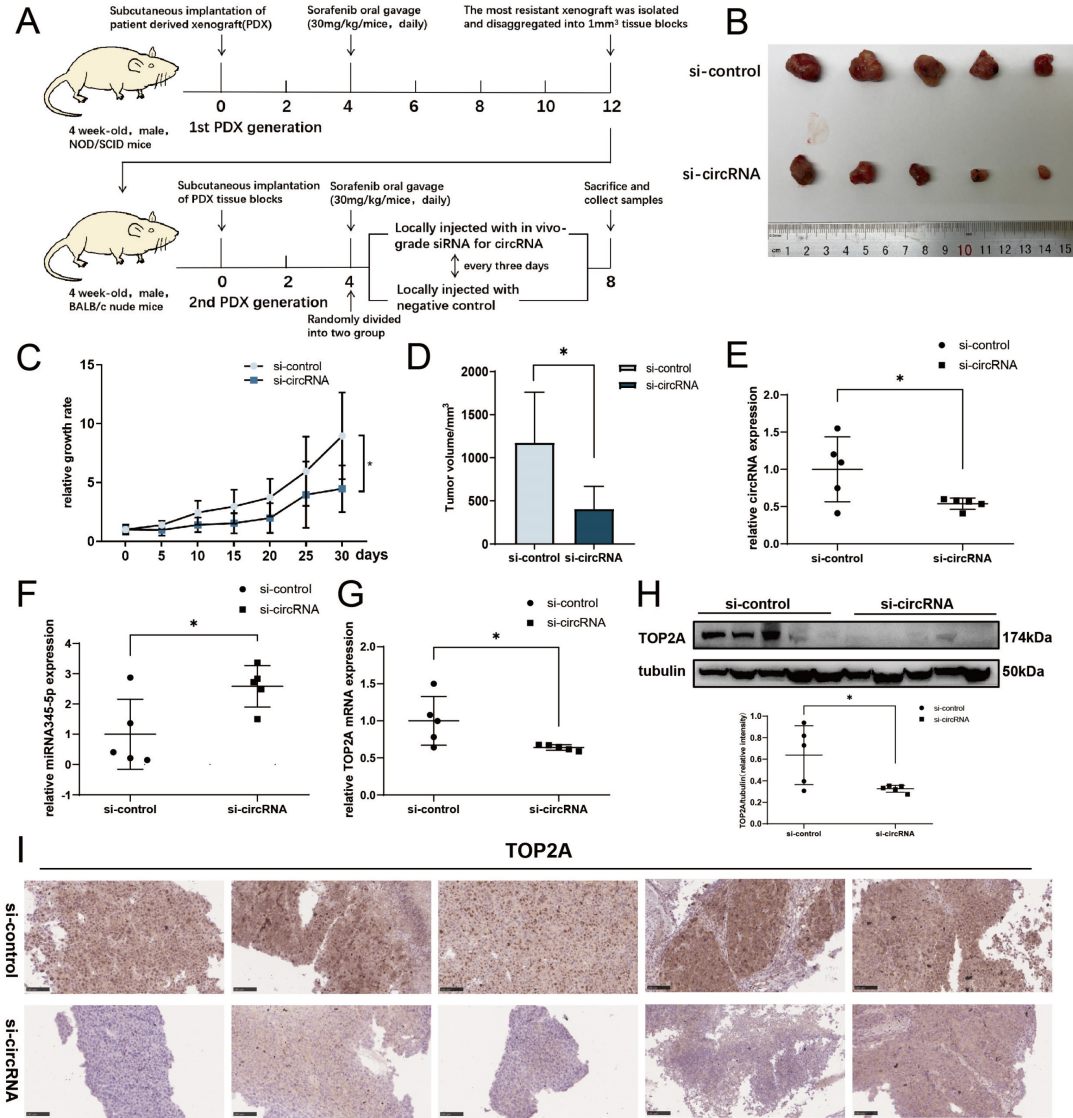
** Knockdown of cDCBLD2 can inhibit HCC sorafenib resistance *in vivo*.** Schematic representation of the establishment of sorafenib-resistant mouse models. The expression levels of cDCBLD2 were reduced in the tumors using siRNA transfection. B. Two groups of subcutaneous xenografts were collected. C. The size of the xenografts *in vivo* was measured every five days using vernier calipers. D. After tumor separation, the size of the xenografts were measured with vernier calipers. E, F, G. The expression levels of cDCBLD2, miR-345-5p, and TOP2A, respectively, were detected by qRT-PCR. H. Western blot analysis of TOP2A protein in the mouse model tumors. I. The protein expression patterns of TOP2A were detected by IHC assays (20×).

To further determine the significance of cDCBLD2 in mediating the resistance of hepatoma cells to sorafenib *in vivo*, we used the sorafenib-resistant patient-derived xenograft (PDX) model **(Fig. 8A)**. In this model, local injection of *in vivo* grade cholesterol-conjugated si-cDCBLD2 around the PDX implantation site resulted in a significantly increased sensitivity to sorafenib treatment compared with injection of a control siRNA** (*P*<0.05, Fig. 8B, C, D)**. In addition, similar to our *in vitro* findings, si-cDCBLD2 treatment in the PDX model resulted in decreased cDCBLD2 **(Fig. 8E)**, increased miR-345-5p **(Fig. 8F)**, and decreased TOP2A mRNA **(Fig. 8G)** expression levels in the tumor. Reduced tumor expression of TOP2A protein was also observed** (Fig. 8H, I)**. In conclusion, these results indicate that knockdown of cDCBLD2 can enhance sensitivity to sorafenib *in vivo*, further supporting the clinical potential of silencing cDCBLD2 to improve the efficacy of sorafenib in drug-resistant HCC patients.

## Discussion

Primary liver cancer is one of the most malignant diseases with high mortality rates in China and worldwide. Each year, the number of new HCC cases and related deaths in China accounts for more than 50% of the world's total 19. Sorafenib, the first-line drug for treating HCC, can improve advanced HCC patient survival rates to a certain extent, but drug resistance remains a significant issue. Understanding the molecular mechanism of sorafenib resistance may provide new therapeutic targets and insights for potential combination therapy approaches for this disease. Although many studies have discussed a possible mechanism 9, 12, 13, 20, 21, no suitable targets or related drugs have been found to overcome sorafenib resistance.

In this study, we investigated a previously understudied circRNA, cDCBLD2, and its role in maintaining sorafenib resistance in HCC. Using a circRNA array with the sorafenib-resistant cell line HepG2-SR we constructed and its corresponding WT cell line, we found that cDCBLD2 was upregulated in sorafenib-resistant HCC cells. Knockdown of cDCBLD2 could significantly enhance the cell killing ability of sorafenib. Notably, local injection of intracholesterol-bound RIG-I si-cDCBLD2 significantly increased liver tumor sensitivity to sorafenib in a mouse model, indicating that siRNA-mediated regulation of cDCBLD2 has good clinical application potential for sorafenib-resistant HCC patients. CircRNAs are ncRNAs characterized by a covalent closed loop structure that provides higher stability relative to linear RNA molecules. Moreover, circRNAs are expressed in tissue-specific and disease stage-specific manners. These properties make circRNAs suitable as therapeutic targets. Many studies have found that circRNAs serve as signaling molecules between cells 22, 23, which also indicates the feasibility of developing drugs that target them. The United States FDA also recently approved the first RNAi treatment drug, Onpattro, for the treatment of polyneuropathy, a peripheral nerve disorder 24. Our research results suggest that a specific siRNA targeting cDCBLD2 can possibly be delivered *in vivo* through arterial chemoembolization or other ways to improve sorafenib efficacy. These findings may lead to a new potential combination therapy strategy for overcoming sorafenib resistance in HCC.

Mechanistically, further experiments showed that cDCBLD2 could bind to miR-345-5p through a ceRNA mechanism and promote TOP2A mRNA stability. CircRNAs can function as ceRNAs that regulate other RNA transcripts by competing for binding of shared miRNAs. Although most miRNAs bind to the 3' UTR of target mRNAs, some studies have shown that certain miRNAs can bind to the CDS region and still result in negative regulation of expression 15. This study discovered a different miRNA binding mode from the traditional classical regulatory mode and validated its role. This can lead to new directions for the currently limited research on miRNAs. With considerable advancements in industrial synthesis technologies, new miRNA-related drugs are frequently emerging. Mirvirasen, an anti-miR (inhibitor)-type drug developed for miR-122, has entered phase II clinical trials for the treatment of HCV infection 25. Many other miRNA drugs have also entered phase I clinical trials 26, 27. In-depth investigations of miRNAs will also support new drug development for HCC, potentially helping overcome the problem of sorafenib resistance encountered in clinical practice.

Importantly, this study demonstrated that a relatively rare occurrence of miRNA-mediated regulation of target gene expression via the mRNA CDS region plays a role. The competitive combination of cDCBLD2 with miR-345-5p resulted in increased TOP2A protein levels, reduced cleavage of apoptotic marker proteins PARP and caspase-3, and a decreased drug killing effect of sorafenib by inhibiting apoptosis in HCC cells. Survival analysis further showed that patients with higher TOP2A levels had poorer OS and RFS rates. Previous studies have reported that TOP2A is characterized by PARP-1 cleavage and caspase-3 activation in gastric cancer, thus triggering the apoptosis pathway 16. TOP2A can also reportedly induce apoptosis in breast cancer through the caspase-3 signaling pathway 17. Our research also confirmed this finding in HCC and verified that TOP2A played a key role in the process of sorafenib resistance in HCC.

Caspase-3 is activated in both exogenous and endogenous apoptotic pathways. Cysteine aspartate proteinase-3 exists in the cytoplasm as an inactive zymogen. Proapoptotic signaling can lead to fragmentation and activation of caspase-3, which subsequently regulates the cleavage of key cell proteins and formation of apoptotic DNA fragments. This thereby amplifies the protease cascade and cleavage reaction, ultimately leading to cell death 28-30. TOP2A plays an important role in many cell processes, such as transcription, DNA replication, and chromatin separation and concentration, and therefore has become a potential target of anticancer drugs 31. TOP2A is also crucial for the survival of proliferating cells because it can entangle DNA through a complete helix through a short double strand break in DNA 32. After irreparable DNA damage, cells undergo apoptosis through several possible mechanisms 33. Activation of PARP-1 and caspase-3 cleavage are markers of apoptosis 34. PARP-1 is activated during DNA damage and plays a role in DNA replication, transcription, and repair. This study found that TOP2A inhibited the caspase-3 dependent apoptosis pathway, which has important implications for many biological pathways.

Some studies also suggest that TOP2A is the key regulatory factor mediating HCC resistance to its second-line treatment drug regorafenib 35. This also reflects the importance of studying TOP2A. At present, doxorubicin, which can selectively inhibit TOP2A, is the most important first-line drug used for general tumor chemotherapy. From our data, we believe doxorubicin treatment may rescue the sensitivity of hepatoma cells resistant to sorafenib, potentially overcoming this acquired drug resistance through a combination therapy approach. The combination of doxorubicin and sorafenib for the treatment of HCC has good clinical prospects, but additional studies are needed to demonstrate its feasibility.

## Conclusions

Taken together, our study confirms that cDCBLD2 can regulate TOP2A expression through miR-345-5p competitively binding to the coding region of TOP2A mRNA, thus affecting both sorafenib resistance and the caspase-3-mediated apoptosis process in HCC cells (Fig. 7). These findings demonstrate that cDCBLD2 and TOP2A are critical for the maintenance of sorafenib resistance in HCC, providing a new strategy and therapeutic targets for overcoming this drug resistance in patients.

## Supplementary Material

Supplementary materials and methods, figures and tables.

## Figures and Tables

**Table 1:** Association between TOP2A expression levels and clinicopathological features in HCC patients.

Variable	TOP2A expression		P value
	Low (n=38) N (%)	High (n=46) N (%)	
Age			0.9101
< 60 years	26 (68.4%)	32 (69.6%)	
≥ 60 years	12 (31.6%)	14 (30.4%)	
Sex			0.3024
Male	33 (86.8%)	43 (93.5%)	
Female	5 (13.2%)	3 (6.5%)	
HBsAg			0.3253
Negative	9 (23.7%)	7 (15.2%)	
Positive	29 (76.3%)	39 (84.8%)	
Cirrhosis			0.2023
No	21 (55.3%)	19 (41.3%)	
Yes	17 (44.7%)	27 (58.7%)	
Tumor size			0.9331
< 5cm	17 (44.7%)	21 (45.7%)	
≥ 5 cm	21 (55.3%)	25 (54.3%)	
Tumor differentiation			0.0000***
Well/moderately	20 (52.6%)	3 (6.5%)	
Poorly	18 (47.4%)	43 (93.5%)	
Tumor thrombi			0.1166
No	27 (71.1%)	25 (54.3%)	
Yes	11 (28.9%)	21 (45.7%)	
Tumor metastasis			0.0162*
No	37 (97.4%)	35 (76.1%)	
Yes	2 (5.3%)	11 (23.9%)	
Recurrence			0.0035**
No	32 (84.2%)	25 (54.3%)	
Yes	6 (15.8%)	21 (45.7%)	

Chi-squared test was employed to determine statistical difference. *p<0.05, **p<0.01, ***p<0.001.

## Abbreviations

HCC: hepatocellular carcinoma
TOP2A: type IIA topoisomerase
PDX: patient-derived xenograft
miR: microRNA
siRNA: small interfering RNA
FDA: United States Food and Drug Administration
TME: Tumor microenvironment
ncRNAs: non-coding RNAs
lncRNAs: long non-coding RNAs
miRNAs: microRNAs
circRNAs: Circular RNAs
ceRNA: competing endogenous RNA
YBX-1: Y-box binding protein 1
m6A: N6-methyladenosine
3' UTR: 3' untranslated region
CDS: coding sequence
qRT-PCR: Quantitative real-time PCR
FISH: Fluorescence *in situ* hybridization
IHC: Immunohistochemical
SEM: standard error of mean
WT: wide type
SR: Sorafenib-resistant
DCBLD2: discoidin, CUB and LCCL domain containing 2
PARP-1: poly ADP-ribose polymerase-1
c-PARP: cleaved-PARP
Cas3: Caspase-3
c-cas3: cleaved-caspase-3
