# cDCBLD2 mediates sorafenib resistance in hepatocellular carcinoma by sponging miR-345-5p binding to the TOP2A coding sequence: Erratum

**DOI:** 10.7150/ijbs.113876

**Published:** 2025-06-16

**Authors:** YeLing Ruan, TianYi Chen, LongBo Zheng, JingWei Cai, Hu Zhao, YaLi Wang, LiYe Tao, JunJie Xu, Lin Ji, XiuJun Cai

**Affiliations:** 1 Key Laboratory of Laparoscopic Technology of Zhejiang Province, Department of General Surgery, Sir Run-Run Shaw Hospital, Zhejiang University School of Medicine - Hangzhou, China.; 2 Zhejiang Minimal Invasive Diagnosis and Treatment Technology Research Center of Severe Hepatobiliary Disease, Zhejiang Research and Development Engineering Laboratory of Minimally Invasive Technology and Equipment - Hangzhou, China.; 3 Zhejiang University Cancer Center - Hangzhou, China.; 4 Liangzhu Laboratory, Zhejiang University Medical Center - Hangzhou, China.; 5 Department of Gastroenterology, The Affiliated Hospital of Qingdao University - Qingdao, China

In our paper, the author noticed the following non subjective errors. We have rechecked the raw data to ensure that the conclusions of the article are not affected by errors. In this regard, all authors agree with the errata, and we apologize for any inconvenience caused by work negligence.

HCCLM3-SR data in Figure 1F should be corrected as follows.

The fourth sentence of the third paragraph in background should be corrected as follows.

Nikolaus Rajewsky and colleagues proposed the functional miRNA sponge model. They identified 63 miR-7 binding sites in CDR1as, a circRNA that inhibits the actions of miR-7^ [^36^]^.

References: 36. Memczak S, Jens M, Elefsinioti A, et al. Circular RNAs are a large class of animal RNAs with regulatory potency. Nature 2013;495:333-8.

Tumor metastasis data in Table 1 should be corrected as follows.

## Figures and Tables

**Figure 1:**
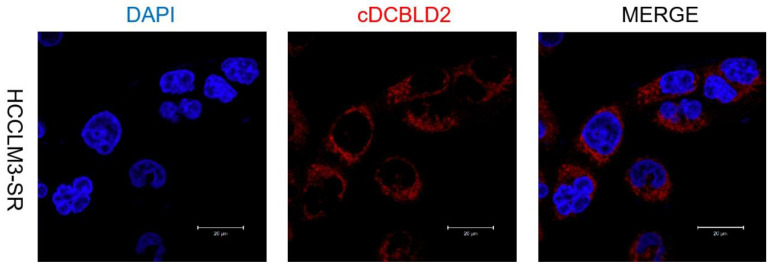
** (F)** Correct image.

**Table 1:** Tumor metastasis data

Variable	TOP2A expression		P value
	Low(n=38) N(%)	High(n=46) N(%)	
Tumor metastasis			0.0187*
No	36(94.7%)	35(76.1%)	
Yes	2(5.3%)	11(23.9%)	

