# Supplementary material for: cDCBLD2 mediates sorafenib resistance in hepatocellular carcinoma by sponging miR-345-5p binding to the TOP2A coding sequence
Source: Int J Biol Sci. 2023 Aug 28;19(14):4608–26. doi: 10.7150/ijbs.86227 (PMC10535695; doi:10.7150/ijbs.86227)
Supplement: Supplementary file 1 — Supplementary materials and methods, figures and tables. [file ijbsv19p4608s1.pdf]

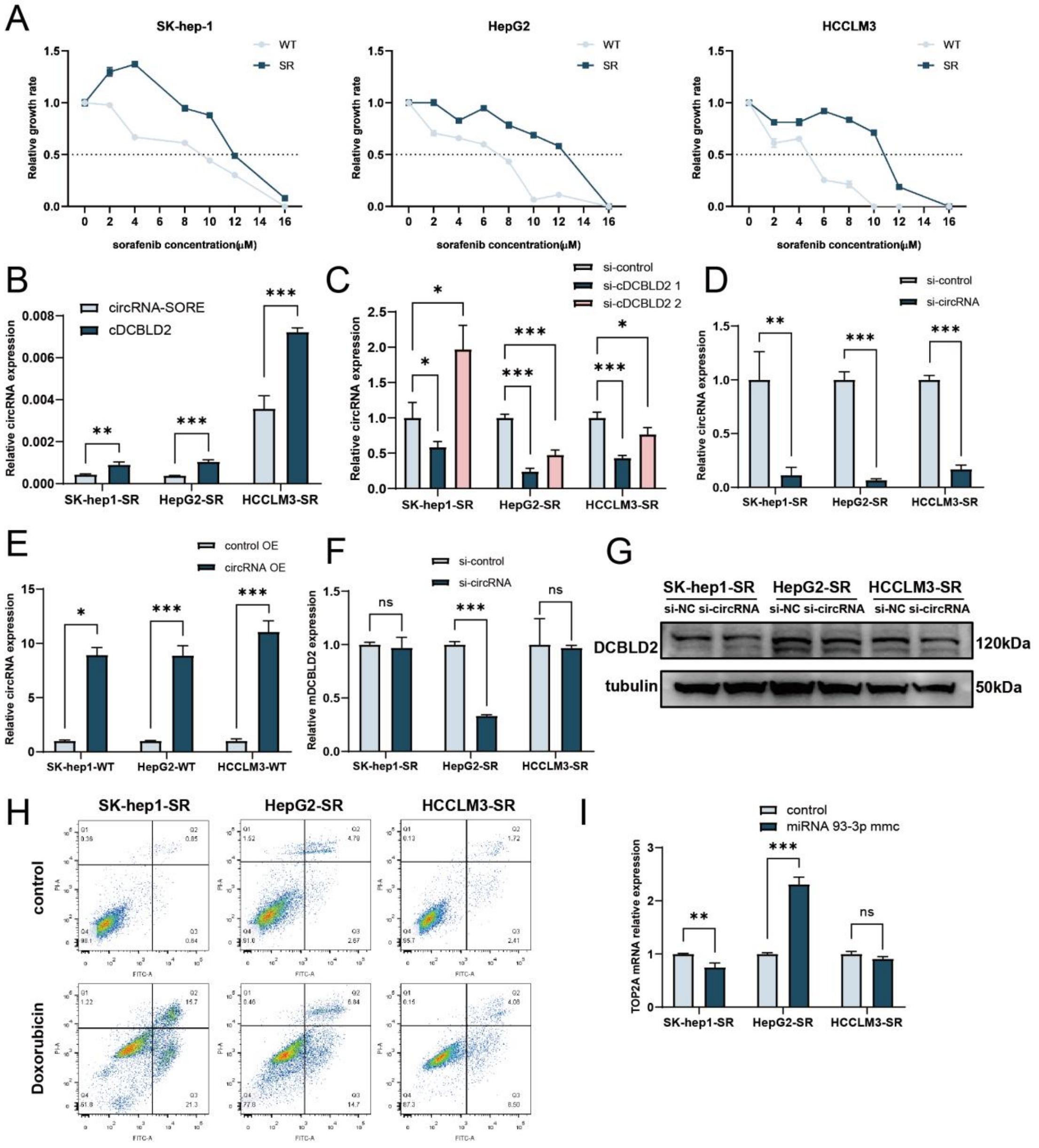

Figure S1.

A. The cells were treated with different concentrations of sorafenib for 48 hours, then the cell viability was measured by CCK-8 assays. The IC50 value of sorafenib-resistant cells was higher than that of wild-type cells. B. Comparison of cDCBLD2 and circRNA-SORE expression levels using qPCR assays. C. qPCR analysis to examine siRNA knockdown efficiency. D,E. Knockdown or overexpression efficiency of cDCBLD2 with sorafenib treatment. F. qPCR results of mDCBLD2 levels after knockdown of cDCBLD2. G. Western blot analysis of DCBLD2 protein levels after knockdown of cDCBLD2. H. Flow cytometry analysis of cells with or without doxorubicin treatment. I. TOP2A mRNA expression patterns after increasing miR-93-3p levels in the three sorafenib-resistant cell lines.

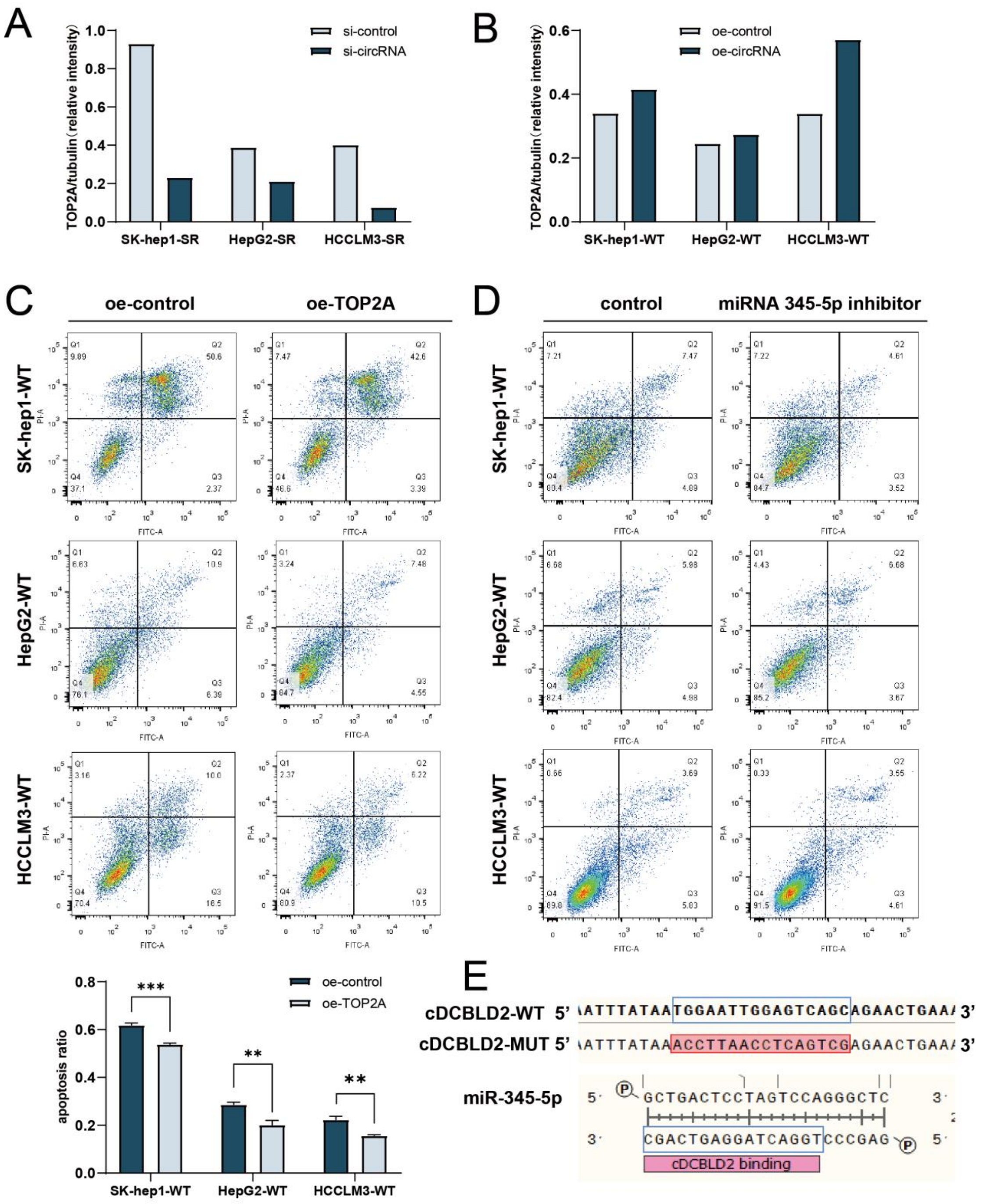

Figure S2.

A,B. Western blot quantitative results for TOP2A protein levels following knockdown or overexpression of cDCBLD2. C. Apoptosis analysis following overexpression of TOP2A. D. Flow cytometry analysis of apoptosis rates after inhibiting miR-345-5p. E. Prediction of cDCBLD2 and miR-345-5p binding sites.

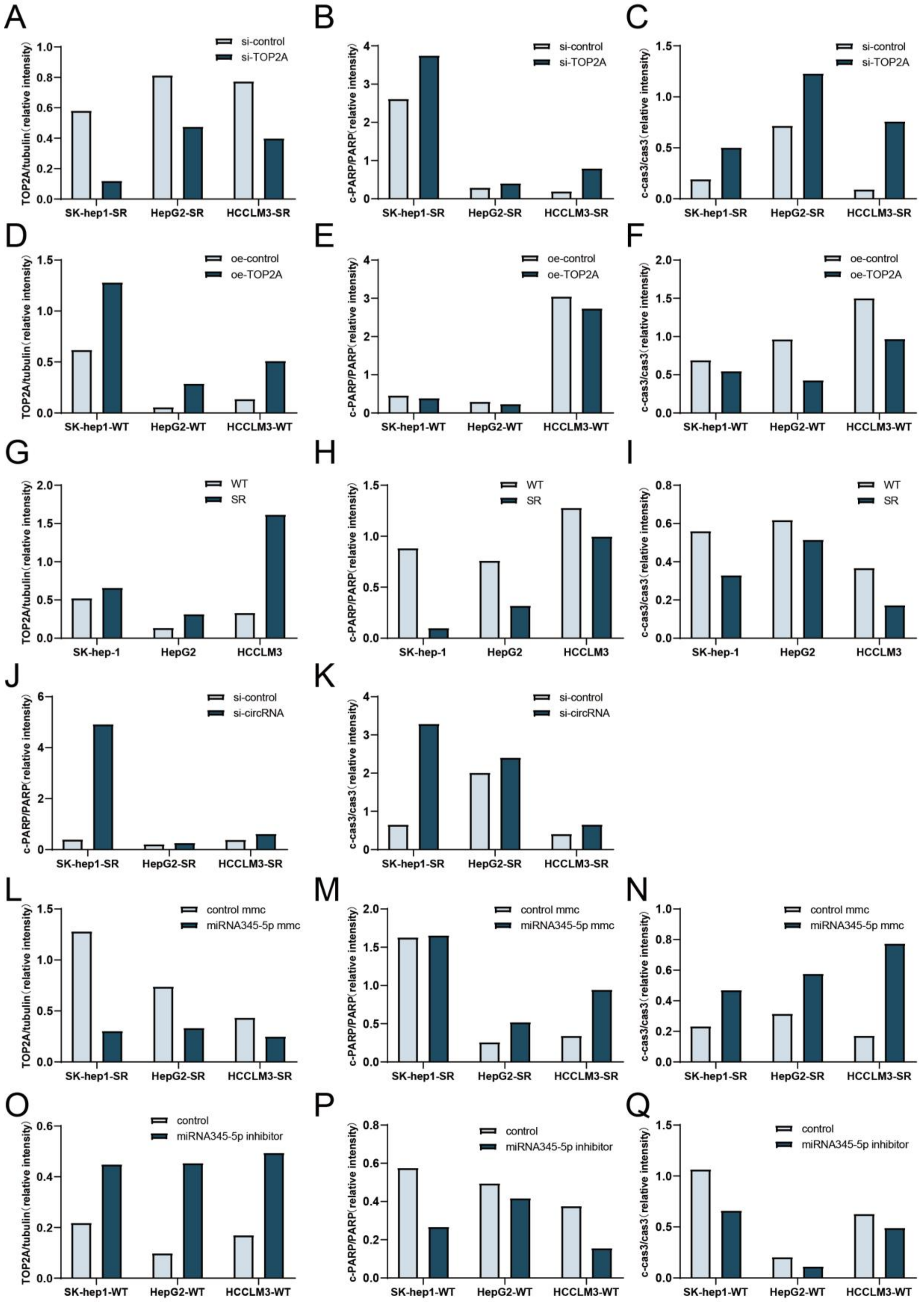

Figure S3.

A–C. Quantitative results of the western blot data in **Fig. 6A**. D–F. Quantitative results of the western blot data in **Fig. 6B**. G–I. Quantitative results of the western blot data in **Fig. 6D**. J–K. Quantitative results of the western blot data in **Fig. 6E**. L–N. Quantitative results of the western blot data in **Fig. 6F**. O–Q. Quantitative results of the western blot data in **Fig. 6G**.

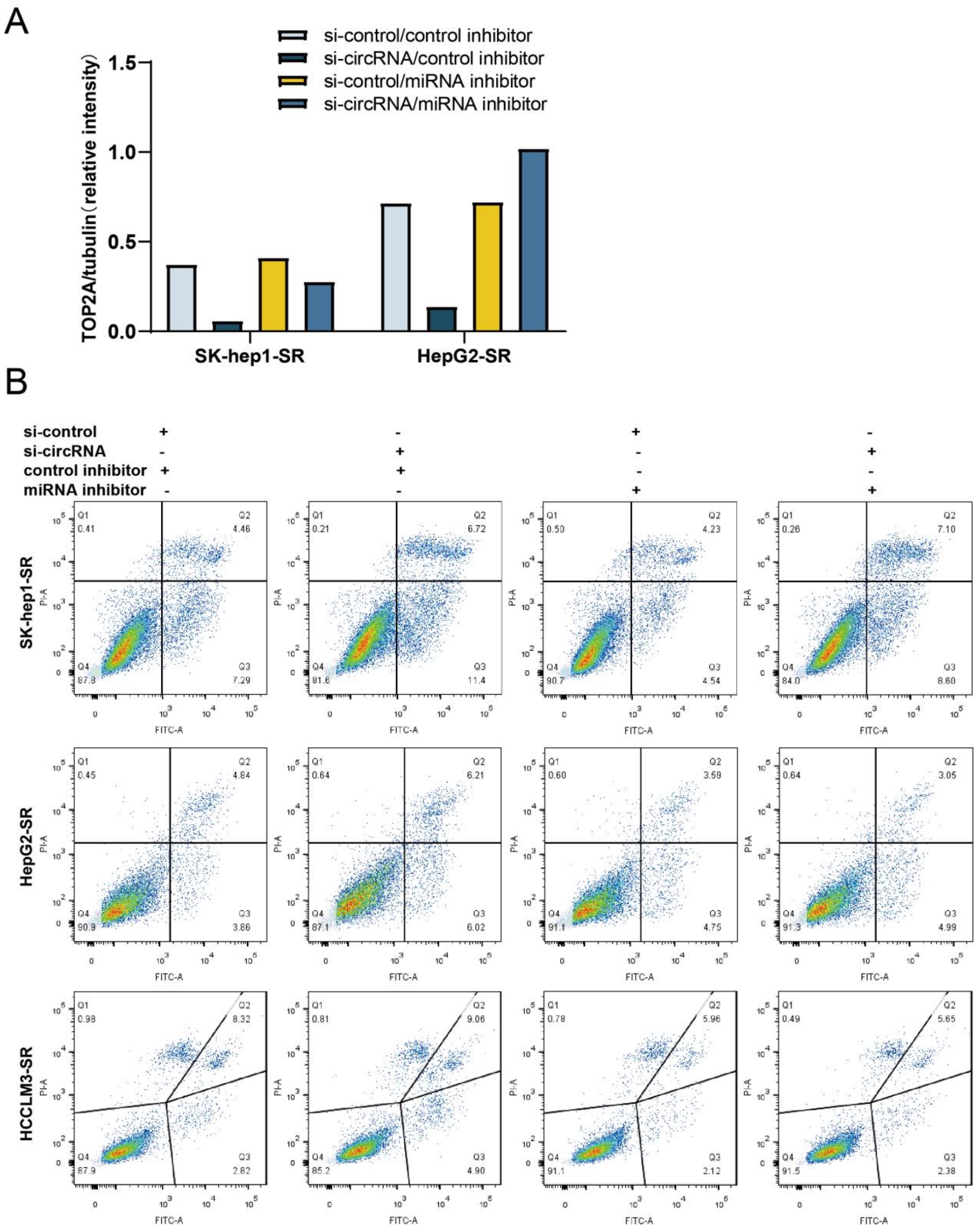

Figure S4.

A. Quantitative results of the western blot data in **Fig. 6I**. B. Flow cytometry analysis of apoptosis data in **Fig. 6K**.

Supplementary information

Materials and Methods

Materials

Sorafenib and Doxorubicin hydrochloride was purchased from MedChemExpress (USA).

Actinomycin D was purchased from Sigma-Aldrich (St. Louis, MO, USA). RNase R was purchased from Epicentre (Madison, WI, USA).

Microarray analysis

The referenced microarray data of circRNAs have been deposited in NCBI's Gene Expression Omnibus (GEO) database (www.ncbi.nlm.nih.gov/geo) under accession number GSE101850.

RNA extraction, reverse transcription, and Quantitative Real-Time PCR (qRT-PCR)

According to the manufacturer's protocol, RNA-Quick purification kit (Esscience, China) or TRIzol reagent (Invitrogen, USA) were used to extract total RNA from cells. Approximately 1μg of RNA was taken from each sample and used to synthesize complementary DNA (cDNA) with Hifair® II 1st Strand cDNA Synthesis SuperMix for qPCR (gDNA digester plus) (Yeasen Biotech, China).

For qRT-PCR, Hieff UNICON® qPCR SYBR Green Master Mix (antibody method, No Rox) (Yeasen Biotech, China) was used, and the analysis was performed using the Loche LightCycler® 480 real-time fluorescent quantitative PCR system (Roche Applied Science, Germany). The relative quantity of mRNA expression was calculated using the ΔCT method.

For miRNA analysis, reverse transcription was carried out using the Hifair® miRNA 1st Strand cDNA Synthesis Kit (A-tail method) (Yeasen Biotech, China), and qPCR was performed using Hieff® miRNA Universal qPCR SYBR Master Mix (Yeasen Biotech, China). The primers were listed below.

Sequences of primers in this study

cDCBLD2	Forward	ATCCATGTTTCTGGACGCGG
	Reverse	GGGCCTAGTACAGTGTGTCC
cDCBLD2 convergent (c)	Forward	TGTGAATGGGAGATCCGTGT
	Reverse	ATTTGCAACCCCAGACCACA
cDCBLD2 divergent (d)	Forward	TGTGGTCTGGGGTTGCAAAT
	Reverse	ACACGGATCTCCCATTCACA
mDCBLD2	Forward (exon3)	CCATGTTTCTGGACGCGGAT
	Reverse (exon5)	CAACCAGCTGGGCAGTACTTA
actin beta	Forward	GGGCATGGGTCAGAAGGATT
	Reverse	TCGATGGGGTACTTCAGGGT
U6	Forward	CTCGCTTCGGCAGCACAT
	Reverse	TTTGCGTGTCATCCTTGCG
GAPDH	Forward	GTCTCCTCTGACTTCAACAGCG
	Reverse	ACCACCCTGTTGCTGTAGCCAA
TTN	Forward	AGCAGGTGCTATCAGTGCTC
	Reverse	GGACAATTGTTGGTGCCTCG
PI15	Forward	GGAAGCGCTACATTTCGCAG
	Reverse	ACACTTTGCCCCGAACTTGA
KIF20B	Forward	GCCAACTTGAATATGGCTAATAGT
	Reverse	AGGCGCAGCATCTTTCTCTT
SPINK5	Forward	AATGCAAAGGATGAGTGCAGTG

	Reverse	AGAACTTTCCATCAGCACCG
TET1	Forward	AGTGGTGACTATGCCAGTGC
	Reverse	CAGACCCCACATCGCTTTCT
WDR35	Forward	ACGTGAAGCTGCAGTGTGTA
	Reverse	TGCATCATCTGTCTGCGTCT
CENPE	Forward	TGGTTGATCTTGCAGGCAGT
	Reverse	TTACAGCCTTCCTTGAGCCG
INSIG1	Forward	ATCTTTTCCTCCGCCTGGTG
	Reverse	TCTCCGAGGTGACTGTCGAT
CAMK4	Forward	CCGGATTACTGGATCGACGG
	Reverse	ATAAGGCTTCTGGGTCCCCT
SERTAD4	Forward	CTCGGAAGGAGCTGCTGAAA
	Reverse	AATGTGATCCTGCCAGTGGG
TOP2A	Forward	GTGTCACCATTGCAGCCTGT
	Reverse	TGTCTGGGCGGAGCAAAATA
U2SURP	Forward	CTGATGGTCAGCGTCGTTCT
	Reverse	TGCGTAATCATCAAGAACACCA
KIF18A	Forward	CACTTGCTGTCCGGGAAGAT
	Reverse	CTGAGGATTTGGGCTGGTGT
hsa-mir-490-5p	Forward	CCATGGATCTCCAGGTGGG
hsa-mir-345-5p	Forward	GCTGACTCCTAGTCCAGGGC
hsa-mir-26b-5p	Forward	GGCGGCTTCAAGTAATTCAGGATAGG
hsa-mir-26a-5p	Forward	GCGGTTCAAGTAATCCAGGATAGGC
hsa-mir-93-3p	Forward	ACTGCTGAGCTAGCACTTCCC
5s	Forward	GAATACCGGGTGCTGTAGGCT

Western blotting analysis

We lysed cells using RIPA buffer (Beyotime Biotechnology, China) supplemented with 5× DualColor Protein Loading Buffer (FDbio Science, China) to acquire protein. The protein was electrophoresed by PAGE gel and blotted onto a polyvinylidene fluoride western blot membrane (Millipore, USA). The PVDF membrane was blocked with TBST containing 5% skimmed milk powder at room temperature for 1 hour. Following the blocking step, PVDF membrane were incubated at 4 ℃ overnight with appropriate antibody. Configure enhanced chemiluminescence reagents (FDbio Science, China) were used to detect antigen-antibody complex on PVDF membrane (FDbio science, China). Grayscale values of the exposed bands were analyzed by Image J. The antibodys were listed below.

Antibody

β-tubulin	FUDE Biological Technology	FD0064
DCBLD2	Cell Signaling Technology	#4804
TOP2A	abcam	ab52934
GAPDH	proteintech	60004-1-Ig
Lamin A/C	Cell Signaling Technology	#4777

PARP	HUABIO	ET1608-56
Cleaved PARP1	abcam	ab32064
caspase3	HUABIO	ET1602-39
Cleaved caspase3	HUABIO	ET1602-47

Cell transfection

Small interfering RNA (siRNA) targeting circRNA_cDCBLD2, TOP2A and controls (si-cDCBLD2, si-TOP2A, si-CTRL) were purchased from Ribobio (Guangzhou, China). MiRNA mimics or inhibitors were purchased from Ribobio (Guangzhou, China). CircRNA_cDCBLD2-PLC5-ciR plasmid and its control were from Geneseed Biotech (Guangzhou, China). TOP2A-GV141 plasmid and its control were from Genechem (Shanghai, China). TOP2A-bind-WT-pmirGLO, TOP2A-bind-mutation1-pmirGLO and TOP2A-bind-mutation2-pmirGLO were from Tsingke Biotechnology (Beijing, China). According to the agreement of the manufacturer, Lipofectamine 3000 reagent (Invitrogen, USA) was used to transiently transfect siRNA, miRNA mimics, miRNA inhibitors or plasmids into HCC cells at working concentration of protocol. After 48~72 hours of transfection, the gene silencing effect was verified through qPCR or Western blot. The specific sequences used in these experiments are listed below.

Sequences of specific targets in this study

si-cDCBLD2 1	Sense (5'-3')	AAACAAGGTGATGGATGTG+dTdT
si-cDCBLD2 2	Sense (5'-3')	GATAAACAAGGTGATGGATG+dTdT
si-TOP2A	Sense (5'-3')	acctttgactctcagacaaaaga+dTdT
hsa-mir-490-5p mimic	Sense (5'-3')	CCAUGGAUCUCCAGGUGGGU
hsa-mir-345-5p mimic	Sense (5'-3')	GCUGACUCCUAGUCCAGGGCUC
hsa-mir-26b-5p mimic	Sense (5'-3')	uucaaguaauucaggauaggu
hsa-mir-26a-5p mimic	Sense (5'-3')	uucaaguaauccaggauaggcu
hsa-mir-93-3p mimic	Sense (5'-3')	ACUGCUGAGCUAGCACUUCCCG
hsa-mir-345-5p inhibitor	Sense (5'-3')	GAGCCCTGGACTAGGAGTCAGC

Relevant plasmid and sequence information:

pmirGLO:

Ttctagttgtttaaacgagctc(SacI)······gtcgac(SalI)ctgcaggcatgcaagctgatccggctgctaacaaagcccgaaaggaagctgagttggctgctgccaccgctgagcaataactagcataaccccttggggcggccgcttcgagcagacatga

PLC5-ciR:

TTTGACCTCCATAGAAGATCTCCACCTCCCAGGTTCAAGCGATTCTCCTCCCTCAGCCTCCCGAGTAGCTGGGACCACAGGCATGCACCACCATCCCCAGCTAATTTTTGCATTATTAGTAGAGTTGGGATTTCTTCACCGTGTTGGCCAGGCCGGTCTTGGACTCCTGACCTCAAGTGATCCAACTGCCTCAGCCTCTCAAAGTGCTAGGATTACAGGGATCTATACTTTTCTGATATTATAAAGATAGTTATCTTCTCCAAGGGAAAAAATCATCTTCATGGAAATTAATTACTTTTTTACAAATTGTGAATTTGACCCTTAAGAGTTTTCTTCCTGATATTTAAAATTGAAAAAAAAATTGTTGACATTAATATTTCTTCTTTCGAATTC(EcoRI)······GGATCC(BamHI)TAGCTAACAACTCCATACTTTTTGGTTGTTTATTAATGTGAAATTTCTGCTAAATGAAATACTTTTGTGTGTGTTTGTGGTAGAAGAGACCACTTCAGTTAAATAAGGAAATCAAGAGAGGATCAATTTAGGAAGATTCAGATATACAGCCGGGTGCAGTGGCTCATGCCTGTAATCCCTGCACTTAGGGAGG

GV141:

CTGGCTAACTAGAGAACCCACTGCTTACTGGCTTATCGAAATTAATACGACTCACTATAGGGAG
ACCCAAGCTGGCTAGCGTTTAAACGGGCCCTCTAGACTCGAG(XhoI)CGCCACC······TCGGTACC(
KpnI)AAGCTTAAGTGACTACAAGGATGACGATGACAAGGATTACAAAGACGACGATGATAAGG
ACTATAAGGATGATGACGACAAATCTAGATAGTTAAACCGCTGATCAGCCTCGACTGTGCCTTC
TAGTTGCCAGCCATCTGTTGTTTGCCCCTC

TOP2A-bind-WT:

Ccctggtctgattcagaatcagataggagcagtgacgaaagtaattttgatgtccctccacgagaaacagagccacggagagc
agcaacaaaaacaaaattcacaatggatttggattcagatgaagatttctcagattttgatgaaaaaactgatgatgaagattttgt
cccatcagatgctagtccacctaagaccaaaacttccccaaaacttagtaacaaagaactgaaaccacagaaaagtgtcgtgtca
gaccttgaagctgatgatgttaagggcagtgtaccactgtcttcaagccctcctgctacacatttcccagatgaaactgaaattaca
aacccagttcctaaaaagaatgtgacagtgaagaagacagcagcaaaaagtcagtcttccacctccactaccggtgccaaaaa
aagggctgccccaaaaggaactaaaagggatccagctttgaattctggtgtctctcaaaagcctgatcctgccaaaaccaagaa
tcgccgcaaaaggaagccatccacttctgatgattctgactctaattttgagaaaattgtttcgaaagcagtcacaagcaagaaat
ccaaggggggagagtgatgacttccatatggactttgactcagctgtggctcctcgggcaaaatctgtacgggcaaagaaacct
ataaagtacctggaagagtcagatgaagatgatctgttttaa

TOP2A-bind-mutation1:

Ccctggtctgattcagaatcagataggagcagtgacgaaagtaattttgatgtccctccacgagaaacactcggtgcctctcgtc
gtacaaaaacaaaattcacaatggatttggattcagatgaagatttctcagattttgatgaaaaaactgatgatgaagattttgtcc
catcagatgctagtccacctaagaccaaaacttccccaaaacttagtaacaaagaactgaaaccacagaaaagtgtcgtgtcag
accttgaagctgatgatgttaagggcagtgtaccactgtcttcaagccctcctgctacacatttcccagatgaaactgaaattacaa
acccagttcctaaaaagaatgtgacagtgaagaagacagcagcaaaaagtcagtcttccacctccactaccggtgccaaaaaa
agggctgccccaaaaggaactaaaagggatccagctttgaattctggtgtctctcaaaagcctgatcctgccaaaaccaagaat
cgccgcaaaaggaagccatccacttctgatgattctgactctaattttgagaaaattgtttcgaaagcagtcacaagcaagaaatc
caaggggggagagtgatgacttccatatggactttgactcagctgtggctcctcgggcaaaatctgtacgggcaaagaaacctat
aaagtacctggaagagtcagatgaagatgatctgttttaa

TOP2A-bind-mutation2:

ccctggtctgattcagaatcagataggagcagtgacgaaagtaattttgatgtccctccacgagaaacagagccacggagagc
agcaacaaaaacaaaattcacaatggatttggattcagatgaagatttctcagattttgatgaaaaaactgatgatgaagattttgt
cccatcagatgctagtccacctaagaccaaaacttccccaaaacttagtaacaaagaactgaaaccacagaaaagtgtcgtgtca
gaccttgaagctgatgatgttaagggcagtgtaccactgtcttcaagccctcctgctacacatttcccagatgaaactgaaattaca
aacccagttcctaaaaagaatgtgacagtgaagaagacagcagcaaaaagtcagtcttccacctccactaccggtgccaaaaa
aagggctgccccaaaaggaactaaaagggatccagctttgaattctggtgtctctcaaaagcctgatcctgccaaaaccaagaa
tcgccgcaaaaggaagccatccacttctgatgattctgactctaattttgagaaaattgtttcgaaagcagtcacaagcaagaaat
ccaaggggggagagtgatgacttccatatggactttgactcagctgtggctcctcgggcaaaatctgtacgggcaaagaaacct
ataatcatggaccttctcagtcttgaagatgatctgttttaa

cDCBLD2:

GTGATGGATGTGGACACACTGTACTAGGCCCTGAGAGTGGAACCCTTACATCCATAAACTACCC
ACAGACCTATCCCAACAGCACTGTTTGTGAATGGGAGATCCGTGTAAAGATGGGAGAGAGAGT
TCGCATCAAATTTGGTGACTTTGACATTGAAGATTCTGATTCTTGTCACTTTAATTACTTGAGAAT
TTATAATGGAATTGGAGTCAGCAGAACTGAAATAGGCAAATACTGTGGTCTGGGGTTGCAAATG
AACCATTCAATTGAATCAAAAGGCAATGAAATCACATTGCTGTTCATGAGTGGAATCCATGTTTC
TGGACGCGGATTTTTGGCCTCATACTCTGTTATAGATAAACAAG

cDCBLD2-MUT:

GTGATGGATGTGGACACACTGTACTAGGCCCTGAGAGTGGAACCCTTACATCCATAAACTACCC
ACAGACCTATCCCAACAGCACTGTTTGTGAATGGGAGATCCGTGTAAAGATGGGAGAGAGAGT
TCGCATCAAATTTGGTGACTTTGACATTGAAGATTCTGATTCTTGTCACTTTAATTACTTGAGAAT
TTATAAACCTTAACCTCAGTCGAGAACTGAAATAGGCAAATACTGTGGTCTGGGGTTGCAAATG
AACCATTCAATTGAATCAAAAGGCAATGAAATCACATTGCTGTTCATGAGTGGAATCCATGTTTC
TGGACGCGGATTTTTGGCCTCATACTCTGTTATAGATAAACAAG

TOP2A CDS:

atggaagtgtcaccattgcagcctgtaaatgaaaatatgcaagtcaacaaaataaagaaaaatgaagatgctaagaaaagact
gtctgttgaaagaatctatcaaaagaaaacacaattggaacatattttgctccgcccagacacctacattggttctgtggaattagt
gacccagcaaatgtgggtttacgatgaagatgttggcattaactatagggaagtcacttttgttcctggtttgtacaaaatctttgat
gagattctagttaatgctgcggacaacaaacaaagggacccaaaaatgtcttgtattagagtcacaattgatccggaaaacaatt
taattagtatatggaataatggaaaaggtattcctgttgttgaacacaaagttgaaaagatgtatgtcccagctctcatatttggac
agctcctaacttctagtaactatgatgatgatgaaaagaaagtgacaggtggtcgaaatggctatggagccaaattgtgtaacat
attcagtaccaaatttactgtggaaacagccagtagagaatacaagaaaatgttcaaacagacatggatggataatatgggaa
gagctggtgagatggaactcaagcccttcaatggagaagattatacatgtatcacctttcagcctgatttgtctaagtttaaaatgc
aaagcctggacaaagatattgttgcactaatggtcagaagagcatatgatattgctggatccaccaaagatgtcaaagtctttctt
aatggaaataaactgccagtaaaaggatttcgtagttatgtggacatgtatttgaaggacaagttggatgaaactggtaactcctt
gaaagtaatacatgaacaagtaaaccacaggtgggaagtgtgtttaactatgagtgaaaaaggctttcagcaaattagctttgtc
aacagcattgctacatccaagggtggcagacatgttgattatgtagctgatcagattgtgactaaacttgttgatgttgtgaagaa
gaagaacaagggtggtgttgcagtaaaagcacatcaggtgaaaaatcacatgtggatttttgtaaatgccttaattgaaaaccc
aacctttgactctcagacaaaagaaaacatgactttacaacccaagagctttggatcaacatgccaattgagtgaaaaatttatca
aagctgccattggctgtggtattgtagaaagcatactaaactgggtgaagtttaaggcccaagtccagttaaacaagaagtgttc
agctgtaaaacataatagaatcaagggaattcccaaactcgatgatgccaatgatgcagggggccgaaactccactgagtgta
cgcttatcctgactgagggagattcagccaaaactttggctgtttcaggccttggtgtggttgggagagacaaatatgggggttttc
cctcttagaggaaaaatactcaatgttcgagaagcttctcataagcagatcatggaaaatgctgagattaacaatatcatcaagat
tgtgggtcttcagtacaagaaaaactatgaagatgaagattcattgaagacgcttcgttatgggaagataatgattatgacagat
caggaccaagatggttcccacatcaaaggcttgctgattaattttatccatcacaactggccctctcttctgcgacatcgttttctgg
aggaatttatcactcccattgtaaaggtatctaaaaacaagcaagaaatggcattttacagccttcctgaatttgaagagtggaag
agttctactccaaatcataaaaaatggaaagtcaaatattacaaaggtttgggcaccagcacatcaaaggaagctaaagaatac
tttgcagatatgaaaagacatcgtatccagttcaaatattctggtcctgaagatgatgctgctatcagcctggcctttagcaaaaaa
cagatagatgatcgaaaggaatggttaactaatttcatggaggatagaagacaacgaaagttacttgggcttcctgaggattac
ttgtatggacaaactaccacatatctgacatataatgacttcatcaacaaggaacttatcttgttctcaaattctgataacgagagat
ctatcccttctatggtggatggtttgaaaccaggtcagagaaaggttttgtttacttgcttcaaacggaatgacaagcgagaagta
aaggttgcccaattagctggatcagtggctgaaatgtcttcttatcatcatggtgagatgtcactaatgatgaccattatcaatttgg
ctcagaattttgtgggtagcaataatctaaacctcttgcagcccattggtcagtttggtaccaggctacatggtggcaaggattctg
ctagtccacgatacatctttacaatgctcagctctttggctcgattgttatttccaccaaaagatgatcacacgttgaagtttttatatg
atgacaaccagcgtgttgagcctgaatggtacattcctattattcccatggtgctgataaatggtgctgaaggaatcggtactgg
gtggtcctgcaaaatccccaactttgatgtgcgtgaaattgtaaataacatcaggcgtttgatggatggagaagaacctttgcca
atgcttccaagttacaagaacttcaagggtactattgaagaactggctccaaatcaatatgtgattagtggtgaagtagctattctt
aattctacaaccattgaaatctcagagcttcccgtcagaacatggacccagacatacaaagaacaagttctagaacccatgttga
atggcaccgagaagacacctcctctcataacagactatagggaataccatacagataccactgtgaaatttgttgtgaagatgac
tgaagaaaaactggcagaggcagagagagttggactacacaaagtcttcaaactccaaactagtctcacatgcaactctatggt
gctttttgaccacgtaggctgtttaaagaaatatgacacggtgttggatattctaagagactttttgaactcagacttaaatattatg
gattaagaaaagaatggctcctaggaatgcttggtgctgaatctgctaaactgaataatcaggctcgctttatcttagagaaaata

gatggcaaaataatcattgaaaataagcctaagaaagaattaattaaagttctgattcagaggggatatgattcggatcctgtga
aggcctggaaagaagcccagcaaaaggttccagatgaagaagaaaatgaagagagtgacaacgaaaaggaaactgaaaa
gagtgactccgtaacagattctggaccaaccttcaactatcttcttgatatgccccttttggtatttaaccaaggaaaagaaagatga
actctgcaggctaagaaatgaaaaagaacaagagctggacacattaaaaagaaagagtccatcagatttgtggaaagaagac
ttggctacatttattgaagaattggaggctgttgaagccaaggaaaaacaagatgaacaagtcggacttcctgggaaagggg
ggaaggccaaggggaaaaaaacacaaatggctgaagttttgccttctccgcgtggtcaaagagtcattccacgaataaccata
gaaatgaaagcagaggcagaaaagaaaaataaaaagaaaattaagaatgaaaatactgaaggaagccctcaagaagatgg
tgtggaactagaaggcctaaaacaaagattagaaaagaaacagaaaagagaaccaggtacaaagacaaagaaacaaacta
cattggcatttaagccaatcaaaaaaggaaagaagagaaatccctggtctgattcagaatcagataggagcagtgacgaaagt
aattttgatgtccctccacgagaaacagagccacggagagcagcaacaaaaacaaaattcacaatggatttggattcagatgaa
gatttctcagattttgatgaaaaaactgatgatgaagattttgtcccatcagatgctagtccacctaagaccaaaacttccccaaaa
cttagtaacaaagaactgaaaccacagaaaagtgtcgtgtcagaccttgaagctgatgatgttaagggcagtgtaccactgtctt
caagccctcctgctacacatttcccagatgaaactgaaattacaaacccagttcctaaaaagaatgtgacagtgaagaagacag
cagcaaaaagtcagtcttccacctccactaccggtgccaaaaaaagggctgccccaaaaggaactaaaagggatccagctttg
aattctggtgtctctcaaaagcctgatcctgccaaaaccaagaatcgccgcaaaaggaagccatccacttctgatgattctgactc
taattttgagaaaattgtttcgaaagcagtcacaagcaagaaatccaagggggagagtgatgacttccatatggactttgactca
gctgtggctcctcgggcaaaatctgtacgggcaaagaaacctataaagtacctggaagagtcagatgaagatgatctgttttaa
